# Unravelling soluble immune checkpoints in chronic lymphocytic leukemia: Physiological immunomodulators or immune dysfunction

**DOI:** 10.3389/fimmu.2022.965905

**Published:** 2022-09-28

**Authors:** Alicia Landeira-Viñuela, Carlota Arias-Hidalgo, Pablo Juanes-Velasco, Miguel Alcoceba, Almudena Navarro-Bailón, Carlos Eduardo Pedreira, Quentin Lecrevisse, Laura Díaz-Muñoz, José Manuel Sánchez-Santos, Ángela-Patricia Hernández, Marina L. García-Vaquero, Rafael Góngora, Javier De Las Rivas, Marcos González, Alberto Orfao, Manuel Fuentes

**Affiliations:** ^1^ Department of Medicine and General Service of Cytometry, Centro de Investigación Biomédica en Red Cáncer (CIBERONC)- CB16/12/00400, Cancer Research Centre-Instituto Universitario de Biología Molecular y Celular del Cáncer (IBMCC), Consejo Superior de Investigaciones Científicas - Universidad de Salamanca (CSIC-USAL), Instituto de Investigación Biomédica de Salamanca (IBSAL), Salamanca, Spain; ^2^ Department of Hematology, University Hospital of Salamanca, Centro de Investigación Biomédica en Red Cáncer (CIBERONC)- CB16/12/00233, Center Research-Centre Instituto Universitario de Biología Molecular y Celular del Cáncer (IBMCC) Consejo Superior de Investigaciones Científicas - Universidad de Salamanca, Instituto de Investigación Biomédica de Salamanca (CSIC-USAL, IBSAL), Salamanca, Spain; ^3^ Systems and Computing Department Instituto Alberto Luiz Coimbra de Pós-Graduação e Pesquisa de Engenharia-Programa de Engenharia de Sistemas e Computação (COPPE-PESC), Universidade Federal do Rio de Janeiro (UFRJ), Rio de Janeiro, Brazil; ^4^ Statistics Department, University of Salamanca, Salamanca, Spain; ^5^ Department of Pharmaceutical Sciences, Organic Chemistry Section, Faculty of Pharmacy, University of Salamanca, Salamanca, Spain; ^6^ Bioinformatics and Functional Genomics Group, Cancer Research Center Instituto Universitario de Biología Molecular y Celular del Cáncer, Consejo Superior de Investigaciones Científicas/Universidad de Salamanca (CIC-IBMCC, CSIC/USAL), Consejo Superior de Investigaciones Científicas (CSIC) and University of Salamanca (USAL), Salamanca, Spain; ^7^ Proteomics Unit, Cancer Research Centre-IBMCC, Instituto de Investigación Biomédica de Salamanca (IBSAL), University of Salamanca-Consejo Superior de Investigaciones Científicas (CSIC), Salamanca, Spain

**Keywords:** soluble immune checkpoints, cytokines profiles, cellular microenvironment, chronic lymphocytic leukaemia (CLL), immune dysfunction

## Abstract

Chronic lymphocytic leukemia (CLL) is a lymphoid neoplasm characterized by the accumulation of mature B cells. The diagnosis is established by the detection of monoclonal B lymphocytes in peripheral blood, even in early stages [monoclonal B-cell lymphocytosis (MBL^hi^)], and its clinical course is highly heterogeneous. In fact, there are well-characterized multiple prognostic factors that are also related to the observed genetic heterogenicity, such as immunoglobulin heavy chain variable region (IGHV) mutational status, del17p, and *TP53* mutations, among others. Moreover, a dysregulation of the immune system (innate and adaptive immunity) has been observed in CLL patients, with strong impact on immune surveillance and consequently on the onset, evolution, and therapy response. In addition, the tumor microenvironment is highly complex and heterogeneous (i.e., matrix, fibroblast, endothelial cells, and immune cells), playing a critical role in the evolution of CLL. In this study, a quantitative profile of 103 proteins (cytokines, chemokines, growth/regulatory factors, immune checkpoints, and soluble receptors) in 67 serum samples (57 CLL and 10 MBL^hi^) has been systematically evaluated. Also, differential profiles of soluble immune factors that discriminate between MBL^hi^ and CLL (sCD47, sCD27, sTIMD-4, sIL-2R, and sULBP-1), disease progression (sCD48, sCD27, sArginase-1, sLAG-3, IL-4, and sIL-2R), or among profiles correlated with other prognostic factors, such as IGHV mutational status (CXCL11/I-TAC, CXCL10/IP-10, sHEVM, and sLAG-3), were deciphered. These results pave the way to explore the role of soluble immune checkpoints as a promising source of biomarkers in CLL, to provide novel insights into the immune suppression process and/or dysfunction, mostly on T cells, in combination with cellular balance disruption and microenvironment polarization leading to tumor escape.

## Introduction

Chronic lymphocytic leukemia (CLL) is one of the most common leukemias in Western countries in adulthood (1). It is characterized by a progressive accumulation of mature B cells of the following phenotypes: CD5^+^, CD19^+^, and CD23^+^. These cells show low levels of surface immunoglobulins (Ig M and Ig D) and clonal expansion in peripheral and secondary lymphoid organs and bone marrow, which are immunologically incompetent (1–3).

Diagnosis of the disease is established by the detection of monoclonal B lymphocytes (B cells) in peripheral blood (≥5 × 10^9^/L), where there is a precursor stage, termed monoclonal B-cell lymphocytosis (MBL^hi^), in which there is a detection of <5 × 10^9^/L clonal B cells with CLL phenotype and absence of CLL-related signs or symptoms (4). The clinical course of CLL is highly heterogeneous; in fact, some patients have stable disease without any treatment, while other patients are suffering from an aggressive form, with relapses or transformations (Richter transformation—a type of non-Hodgkin lymphoma called fast-growing diffuse large B-cell lymphoma), and who need early treatment (5, 6). Relevant prognostic biomarkers for CLL include age, immunoglobulin heavy chain variable region mutational status (IGHV—mutated or unmutated), cytogenetic profiles (del17p, del11q, trisomy 12, or del13q) and gene mutations (*TP53*, *NOTCH1*, *SF3B1*, *ATM*, and *MYD88*, among others). Among all of them, the following biomarkers are associated with good prognosis: age (<65 years), mutated IGHV, del13q, and mutated MYD88. On the other hand, the following are associated with poor prognosis: unmutated IGHV, del17p, del11q, mutations on *NOCTH1, SF3B1,TP53*, and *ATM.* Currently, the therapeutic algorithm used to establish the type of treatment is based on IGHV mutational status, *TP53* mutations, and del17p as key prognostic biomarkers (7, 8).

CLL is characterized by a strong correlation with an intense alteration of the immune system (innate and adaptive response) with a strong impact on immune surveillance, which is interconnected and highly relevant in onset, evolution and therapeutic response it has been reported altered immunological functions (1, 9). In this regard, multiple studies have analysed the role of T-cells (Th1, Th2 and Tregs), nurse-like cells, dendritic cells, or bone marrow stromal cells in tumor immune surveillance and tumor pathogenesis (2, 10).

Many alterations in the balance of immunomodulators and soluble immune factors could support the growth of the leukemic clone. Some biomolecules (i.e., cytokines, chemokines, and immune checkpoints) have been reported to affect the life of B-CLL cells *in vivo*, by both stimulation of growth and defense against programmed cell death (1, 10, 11).

Also, the importance of the tumor microenvironment (TME) in the immune suppression and CLL development is becoming more relevant (12). It has been observed that B-CLL cells when cultured *in vivo*, without the presence of other cell types, exhibit spontaneous death; this action is inhibited when cytokines and accessory cells are presented. This confirms the complexity of the intercellular and intermolecular interactions to which these tumor cells are exposed (1, 4, 7). These include microenvironmental compartments (lymph nodes and bone marrow) where B-CLL cells receive proliferative and survival signals. In these niches, B-CLL cells establish close and intimate interactions with the matrix and multiple cell types (i.e., fibroblasts, immune response cells, and endothelial cells), generating a bidirectional network that ensures contact through effector molecules that can be expressed or secreted (adhesion molecules, cell surface ligands, chemokines, cytokines, and receptors, among others). This leads to the manipulation and alteration of cytokine balance of the microenvironment by B-CLL cells, enabling disease development and progression, as well as drug resistance (2, 4, 5, 7, 10, 13).

Moreover, B-CLL cells acquire the capacity to evade the immune response, taking advantage of immune checkpoint pathways due to increased levels of inhibitory proteins such as PD-1, PD-L1, CTLA-4, TIM-3, LAG-3, and CD47, and are responsible for modulating the activity of T and NK cells by playing a negative role in their activation. In addition, the B-CLL cells themselves act as antigen-presenting cells, but in this case, they reduce the surface expression of HLA molecules, which allows them to be weakly immunogenic (12, 14, 15). Also, several studies report that B-CLL cells express signals to inhibit phagocytosis capacity of the macrophages. In fact, high levels of antiphagocytic molecules like PD-L1, major histocompatibility complex I (MHC-I), CD24, and CD47 allow the inhibition of macrophage action and the breaking of immune homeostasis, which leads to the persistence of tumoral cells (16, 17).

Currently, it is well-known that immune checkpoints have non-redundant functions, although the same checkpoint is susceptible to several ligands. Hence, depending on the type of binding, it can have positive or negative regulation on the lymphocytes (15, 18). In addition to immune checkpoints, it is important to consider the co-existence with cytokines, which can have a pleiotropic effect and redundancy of functions (15, 19). Bearing this in mind, in this study, 103 proteins’ soluble isoforms (cytokines, chemokines, growth/regulatory factors, immune checkpoints, and soluble receptors) in plasma from CLL patients were simultaneously analyzed at different stages of the disease and at the pre-stage (MBL^hi^) to decipher profiles related to TME, immune dysfunction, and disease prognosis.

## Materials and methods

### Patients

Sixty-seven plasma samples from 57 CLL diagnostic adults and 10 MBL^hi^ diagnostic adults were collected between May 2018 and October 2020 (29 women and 38 men, median age of 70 years, ranging from 36 to 91 years) (**Table 1** and **Supplementary Table 1**). The diagnosis was made according to the National CLL Guidelines of the Spanish CLL Group (GELLC) based on the International Workshop on Chronic Lymphocytic Leukemia (iwCLL) (20). Staging was performed according to Binet and Rai criteria (21, 22). Informed consent was given by each individual before entering the study, and approved by the local ethics committee of the University Hospital of Salamanca (HUS, Salamanca, Spain). In all cases, peripheral blood (PB) samples (10 ml/case) were obtained in EDTA-coated tubes. Immediately after collection, PB samples were centrifuged to 800 g for 10 min at RT, and the plasma was stored at −80°C until analysis.

**Table 1:** Clinical and biological characteristics of patient cohort.

Clinical Information		Frequency (*n*)		Percentage (%)	
**Gender**	Female	29		43.3	
	Male	38		56.7	
**Age**	≤65	24		35.8	
	>65	43		64.2	
**Diagnosis**	MBL^hi^	10		14.9	
	CLL	57		85.1	
**CLL status**	Stable/constant	42		73.7	
	Progression	15		26.3	
**Binet stage**	A	54		80.6	
	B	6		9	
	C	7		10.4	
**Rai stage**	0	45		67.2	
	I	5		7.5	
	II	10		14.9	
	III	1		1.5	
	IV	6		9	
**Treatment status**	Previously to 1st line	9		60	
	Time from 1st line	5		33.3	
**IGHV gene status**	Mutated	45		67.2	
	Unmutated	22		32.8	
**Cytogenetic**	Normal	24		35.8	
	Abnormal	43		64.2	
**Total**		67		100	

### Sample processing

One milliliter of each plasma sample was defrosted on ice and centrifuged for 5 min to 2000 g. One hundred microliters of each sample was aliquoted into 96-well polypropylene PCR microplates (Axygen, EE.UU.). Aliquots in 96-well plates were stored at −80°C until use.

### Immune monitoring

In this study, all serum samples have been analyzed by use of Luminex technology. All Luminex kits (Luminex Inc, EE.UU.) used for this study are described in detail in **Table 2**.

**Table 2:** List of soluble immune factors in the study.

Group	Target	Assay	UniProt ID	Gene	Symbol protein
1	Cytokine	Human Immune Monitoring 65-plex ProcartaPlex Panel for MAGPIX	P09919	CSF3	G-CSF
1	Cytokine	Human Immune Monitoring 65-plex ProcartaPlex Panel for MAGPIX	P04141	CSF2	GM-CSF
1	Chemokine	Human Immune Monitoring 65-plex ProcartaPlex Panel for MAGPIX	P09341	CXCL1	CXCL1/Gro α
1	Growth/regulatory factor	Human Immune Monitoring 65-plex ProcartaPlex Panel for MAGPIX	P14210	HGF	HGF
1	Cytokine	Human Immune Monitoring 65-plex ProcartaPlex Panel for MAGPIX	P48551	IFNAR2	IFN α
1	Cytokine	Human Immune Monitoring 65-plex ProcartaPlex Panel for MAGPIX	P01579	IFNG	IFN γ
1	Cytokine	Human Immune Monitoring 65-plex ProcartaPlex Panel for MAGPIX	P01583	IL1A	IL-1α
1	Cytokine	Human Immune Monitoring 65-plex ProcartaPlex Panel for MAGPIX	P01584	IL1B	IL-1β
1	Cytokine	Human Immune Monitoring 65-plex ProcartaPlex Panel for MAGPIX	P60568	IL2	IL-2
1	Soluble receptor	Human Immune Monitoring 65-plex ProcartaPlex Panel for MAGPIX	P31785	IL2RG	sIL-2R
1	Cytokine	Human Immune Monitoring 65-plex ProcartaPlex Panel for MAGPIX	P08700	IL3	IL-3
1	Cytokine	Human Immune Monitoring 65-plex ProcartaPlex Panel for MAGPIX	P05112	IL4	IL-4
1	Cytokine	Human Immune Monitoring 65-plex ProcartaPlex Panel for MAGPIX	P05113	IL5	IL-5
1	Cytokine	Human Immune Monitoring 65-plex ProcartaPlex Panel for MAGPIX	P05231	IL6	IL-6
1	Cytokine	Human Immune Monitoring 65-plex ProcartaPlex Panel for MAGPIX	P13232	IL7	IL-7
1	Cytokine	Human Immune Monitoring 65-plex ProcartaPlex Panel for MAGPIX	P10145	CXCL8	IL-8
1	Cytokine	Human Immune Monitoring 65-plex ProcartaPlex Panel for MAGPIX	P15248	IL9	IL-9
1	Cytokine	Human Immune Monitoring 65-plex ProcartaPlex Panel for MAGPIX	P22301	IL10	IL-10
1	Cytokine	Human Immune Monitoring 65-plex ProcartaPlex Panel for MAGPIX	P29460/P29459	IL12β/IL12α	IL-12p70
1	Cytokine	Human Immune Monitoring 65-plex ProcartaPlex Panel for MAGPIX	P35225	IL13	IL-13
1	Cytokine	Human Immune Monitoring 65-plex ProcartaPlex Panel for MAGPIX	P40933	IL15	IL-15
1	Cytokine	Human Immune Monitoring 65-plex ProcartaPlex Panel for MAGPIX	Q14005	IL16	IL-16
1	Cytokine	Human Immune Monitoring 65-plex ProcartaPlex Panel for MAGPIX	Q16552	IL17A	IL-17A
1	Cytokine	Human Immune Monitoring 65-plex ProcartaPlex Panel for MAGPIX	Q14116	IL18	IL-18
1	Cytokine	Human Immune Monitoring 65-plex ProcartaPlex Panel for MAGPIX	Q9NYY1	IL20	IL-20
1	Cytokine	Human Immune Monitoring 65-plex ProcartaPlex Panel for MAGPIX	Q9HBE4	IL21	IL-21
1	Cytokine	Human Immune Monitoring 65-plex ProcartaPlex Panel for MAGPIX	Q9GZX6	IL22	IL-22
1	Cytokine	Human Immune Monitoring 65-plex ProcartaPlex Panel for MAGPIX	Q9NPF7	IL23A	IL-23
1	Cytokine	Human Immune Monitoring 65-plex ProcartaPlex Panel for MAGPIX	Q8NEV9	IL27	IL-27
1	Cytokine	Human Immune Monitoring 65-plex ProcartaPlex Panel for MAGPIX	Q6EBC2	IL31	IL-31
1	Chemokine	Human Immune Monitoring 65-plex ProcartaPlex Panel for MAGPIX	P02778	CXCL10	CXCL10/IP-10
1	Chemokine	Human Immune Monitoring 65-plex ProcartaPlex Panel for MAGPIX	O14625	CXCL11	CXCL11/I-TAC
1	Cytokine	Human Immune Monitoring 65-plex ProcartaPlex Panel for MAGPIX	P15018	LIF	LIF
1	Chemokine	Human Immune Monitoring 65-plex ProcartaPlex Panel for MAGPIX	P13500	CCL2	CCL2/MCP-1
1	Chemokine	Human Immune Monitoring 65-plex ProcartaPlex Panel for MAGPIX	P80075	CCL8	CCL8/MCP-2
1	Chemokine	Human Immune Monitoring 65-plex ProcartaPlex Panel for MAGPIX	P80098	CCL7	CCL7/MCP-3
1	Cytokine	Human Immune Monitoring 65-plex ProcartaPlex Panel for MAGPIX	P09603	CSF1	M-CSF
1	Chemokine	Human Immune Monitoring 65-plex ProcartaPlex Panel for MAGPIX	O00626	CCL22	CCL22/MDC
1	Cytokine	Human Immune Monitoring 65-plex ProcartaPlex Panel for MAGPIX	P14174	MIF	MIF
1	Chemokine	Human Immune Monitoring 65-plex ProcartaPlex Panel for MAGPIX	Q07325	CXCL9	CXCL9/MIG
1	Chemokine	Human Immune Monitoring 65-plex ProcartaPlex Panel for MAGPIX	P10147	CCL3	CCL3/MIP-1α
1	Chemokine	Human Immune Monitoring 65-plex ProcartaPlex Panel for MAGPIX	P13236	CCL4	CCL4/MIP-1β
1	Chemokine	Human Immune Monitoring 65-plex ProcartaPlex Panel for MAGPIX	P78556	CCL20	CCL20/MIP-3α
1	Growth/regulatory factor	Human Immune Monitoring 65-plex ProcartaPlex Panel for MAGPIX	P03956	MMP1	MMP-1
1	Growth/regulatory factor	Human Immune Monitoring 65-plex ProcartaPlex Panel for MAGPIX	P21583	KITLG	SCF
1	Chemokine	Human Immune Monitoring 65-plex ProcartaPlex Panel for MAGPIX	P48061	CXCL12	CXCL12/SDF-1α
1	Cytokine	Human Immune Monitoring 65-plex ProcartaPlex Panel for MAGPIX	P01375	TNF	TNF-α
1	Cytokine	Human Immune Monitoring 65-plex ProcartaPlex Panel for MAGPIX	P01374	LTA	TNF-β
1	Soluble receptor	Human Immune Monitoring 65-plex ProcartaPlex Panel for MAGPIX	P20333	TNFRSF1B	sTNF-R2
1	Soluble receptor	Human Immune Monitoring 65-plex ProcartaPlex Panel for MAGPIX	O14798	TNFRSF10C	sTRAIL
1	Cytokine	Human Immune Monitoring 65-plex ProcartaPlex Panel for MAGPIX	Q969D9	TSLP	TSLP
1	Chemokine	Human Immune Monitoring 65-plex ProcartaPlex Panel for MAGPIX	O43927	CXCL13	CXCL13/BLC
1	Growth/regulatory factor	Human Immune Monitoring 65-plex ProcartaPlex Panel for MAGPIX	P01138	NGF	β-NGF
1	Soluble receptor	Human Immune Monitoring 65-plex ProcartaPlex Panel for MAGPIX	P28908	TNFRSF8	sCD30
1	Soluble receptor	Human Immune Monitoring 65-plex ProcartaPlex Panel for MAGPIX	P29965	CD40LG	sCD40L
1	Chemokine	Human Immune Monitoring 65-plex ProcartaPlex Panel for MAGPIX	P42830	CXCL5	CXCL5/ENA-78
1	Chemokine	Human Immune Monitoring 65-plex ProcartaPlex Panel for MAGPIX	P51671	CCL11	CCL11/Eotaxin
1	Chemokine	Human Immune Monitoring 65-plex ProcartaPlex Panel for MAGPIX	O00175	CCL24	CCL24/Eotaxin-2
1	Chemokine	Human Immune Monitoring 65-plex ProcartaPlex Panel for MAGPIX	Q9Y258	CCL26	CCL26/Eotaxin-3
1	Growth/regulatory factor	Human Immune Monitoring 65-plex ProcartaPlex Panel for MAGPIX	P09038	FGF2	FGF-2
1	Chemokine	Human Immune Monitoring 65-plex ProcartaPlex Panel for MAGPIX	P78423	CX3CL1	CX3CL1/Fractalkine
1	Growth/regulatory factor	Human Immune Monitoring 65-plex ProcartaPlex Panel for MAGPIX	P15692	VEGFA	VEGF-A
1	Soluble receptor	Human Immune Monitoring 65-plex ProcartaPlex Panel for MAGPIX	O75888	TNFSF13	sAPRIL
1	Soluble receptor	Human Immune Monitoring 65-plex ProcartaPlex Panel for MAGPIX	Q9Y275	TNFSF13B	sBAFF/sBLYS
1	Soluble receptor	Human Immune Monitoring 65-plex ProcartaPlex Panel for MAGPIX	O43508	TNFSF12	sTWEAK
2	I.Check LT	Human Immuno-Oncology Checkpoint Marker Panel	Q9Y5U5	TNFRSF18	sGITR
2	I.Check LT	Human Immuno-Oncology Checkpoint Marker Panel	O43557	TNFSF14	sHVEM
2	I.Check LT	Human Immuno-Oncology Checkpoint Marker Panel	P10747	CD28	sCD28
2	I.Check LT	Human Immuno-Oncology Checkpoint Marker Panel	P33681	CD80	sCD80/sB7-1
2	I.Check LT	Human Immuno-Oncology Checkpoint Marker Panel	Q07011	TNFRSF9	s4-1BB/sCD137
2	I.Check LT	Human Immuno-Oncology Checkpoint Marker Panel	P26842	CD27	sCD27
2	I.Check LT	Human Immuno-Oncology Checkpoint Marker Panel	P16410	CTLA4	sCD152/sCTLA4
2	I.Check LT	Human Immuno-Oncology Checkpoint Marker Panel	Q15116	PDCD1	sPD1
2	I.Check LT	Human Immuno-Oncology Checkpoint Marker Panel	Q9NZQ7	CD274	sPD-L1
2	I.Check LT	Human Immuno-Oncology Checkpoint Marker Panel	Q9BQ51	PDCD1LG2	sPD-L2
2	I.Check LT	Human Immuno-Oncology Checkpoint Marker Panel	P14902	IDO1	sIDO
2	I.Check LT	Human Immuno-Oncology Checkpoint Marker Panel	Q7Z6A9	BTLA	sBTLA
2	I.Check LT	Human Immuno-Oncology Checkpoint Marker Panel	P18627	LAG-3	sLAG-3
2	I.Check LT	Human Immuno-Oncology Checkpoint Marker Panel	Q8TDQ0	HAVCR2	sTIM-3
2	I.Check LT	ProcartaPlex Human Immuno-Oncology Checkpoint Panel 3	P23510	TNFSF4	sCD134/sOX40
2	I.Check LT	ProcartaPlex Human Immuno-Oncology Checkpoint Panel 3	Q5ZPR3	CD276	sCD276/sB7-H3
2	I.Check LT	ProcartaPlex Human Immuno-Oncology Checkpoint Panel 3	Q08722	CD47	sCD47/sIAP
2	I.Check LT	ProcartaPlex Human Immuno-Oncology Checkpoint Panel 3	P09326	CD48	sCD48/sBLAST-1
2	I.Check LT	ProcartaPlex Human Immuno-Oncology Checkpoint Panel 3	O00182	LGALS9	sGalectin-9
2	I.Check LT	ProcartaPlex Human Immuno-Oncology Checkpoint Panel 3	O75144	ICOSLG	sICOS Ligand/sB7-H2
2	I.Check LT	ProcartaPlex Human Immuno-Oncology Checkpoint Panel 3	Q96H15	TIMD4	sTIMD-4
2	I.Check LT	ProcartaPlex Human Immuno-Oncology Checkpoint Panel 3	Q9H7M9	VSIR	sVISTA/sB7-H5
3	I.Check NK	ProcartaPlex Human Immuno-Oncology Checkpoint Panel 2	Q29983	MICA	sMICA
3	I.Check NK	ProcartaPlex Human Immuno-Oncology Checkpoint Panel 2	Q29980	MICB	sMICB
3	I.Check NK	ProcartaPlex Human Immuno-Oncology Checkpoint Panel 2	Q9BZM6	ULBP1	sULBP-1
3	I.Check NK	ProcartaPlex Human Immuno-Oncology Checkpoint Panel 2	Q9BZM4	ULBP3	sULBP-3
3	I.Check NK	ProcartaPlex Human Immuno-Oncology Checkpoint Panel 2	Q8TD07	RAET1E	sULBP-4
3	I.Check NK	ProcartaPlex Human Immuno-Oncology Checkpoint Panel 2	P40200	CD96	sCD96/sTactile
3	I.Check NK	ProcartaPlex Human Immuno-Oncology Checkpoint Panel 2	P15151	PVR	sCD155/sPVR
3	I.Check NK	ProcartaPlex Human Immuno-Oncology Checkpoint Panel 2	Q92692	NECTIN2	sCD112/sNectin-2
3	I.Check NK	ProcartaPlex Human Immuno-Oncology Checkpoint Panel 2	P21589	NT5E	sCD73/sNT5E
3	I.Check NK	ProcartaPlex Human Immuno-Oncology Checkpoint Panel 2	P05089	ARG1	sArginase-1
3	I.Check NK	ProcartaPlex Human Immuno-Oncology Checkpoint Panel 2	Q9Y286	SIGLEC7	sSiglec-7
3	I.Check NK	ProcartaPlex Human Immuno-Oncology Checkpoint Panel 2	Q9Y336	SIGLEC9	sSiglec-9
3	I.Check NK	ProcartaPlex Human Immuno-Oncology Checkpoint Panel 2	P14222	PRF1	sPerforin
3	I.Check NK	ProcartaPlex Human Immuno-Oncology Checkpoint Panel 2	P12830	CDH1	sE-Cadherin
3	I.Check NK	ProcartaPlex Human Immuno-Oncology Checkpoint Panel 3	Q68D85	NCR3LG1	sB7-H6
3	I.Check NK	ProcartaPlex Human Immuno-Oncology Checkpoint Panel 3	P05109	S100A8	sS100A8
3	I.Check NK	ProcartaPlex Human Immuno-Oncology Checkpoint Panel 3	P06702	S100A9	sS100A9

I.Check is immune checkpoint.

The Luminex system used microspheres or a bead set marker with different ratios of two different fluorophores, conjugated with monoclonal antibodies specific for different cytokines, chemokines, immune checkpoints, growth/regulatory factors, and soluble receptors. In the assay, once the protein of interest is bound, it is incubated with a secondary detection antibody specific for the molecule of interest. The color-coded beads are read on a MAGPIX^®^ system (Luminex Corporation, EE.UU.), which has two lasers, one that can identify the bead, and thus the protein of interest, and one that can detect the quantity of the detection agent on the bead, and thus the quantity of the soluble protein of interest (i.e., cytokine, chemokine, and soluble immune checkpoints). In all the analysis, the MAGPIX^®^ reproducibility has been evaluated by calibration and verification reagents as described by the manufactures (MPXIVD-CAL-K25 and MPXIVD-PVER-K25, respectively). In addition, a standard curve has been added to the plate in duplicate for each experiment. Following the generation of a five-parameter logistic curve, the standard recovery was calculated using the following equation: (observed concentration/expected concentration) × 100. A recovery range between 70% and 130% is recommended by the manufacturer. Any sample that fell on an area of the curve that was outside these ranges was not considered accurate. A positive sample is considered if it was above the limits of detection as determined by the manufacturer descriptions.

#### Cytokines, chemokines, and growth factor profiles in serum

Here, the Luminex kit used is “Human Monitoring 65-plex” ProcartaPlex Panel for MAGPIX^®^ (EPX650-16500-091). All the serum samples were incubated in a 96-well Solid Polystyrene Microplate (Corning^®^, EE.UU.), and during the washing process, a magnetic 96-well separator (Thermo Fisher Scientific, EE.UU.) was used following the manufacturer’s instructions. The acquisition was performed on MAGPIX^®^ Instrument and xMAP^®^ component software v. 4.2. All experiments were performed by the same operator according to the manufacturer’s instructions.

#### Soluble immune checkpoint profiles in serum

These studies were performed with the following Luminex kits according to the manufacturer’s instructions: Human Immuno-Oncology Checkpoint marker Panel 1 (EPX14A-15803-901), Human Immuno-Oncology Checkpoint marker Panel 2 (EPX140-15815-901), and Human Immuno-Oncology Checkpoint marker Panel 3 (EPX-100-1582-901). All the serum samples were incubated in a 96-well Solid Polystyrene Microplate (Corning^®^, EE.UU.), and during the washing process, a magnetic 96-well separator (Thermo Fisher Scientific, EE.UU.) was used following the manufacturer’s instructions. The acquisition was performed on MAGPIX^®^ Instrument and xMAP^®^ component software v. 4.2. All experiments were performed by the same operator according to the manufacturer’s instructions.

#### Quantitative analysis

For each protein (contained in **Table 2**), the concentration (pg/ml) was determined by a five-parameter logistic (5PL) curve-fitting algorithm, which was used for concentration data and whose equation is as follows:


$$y=a+\frac{b-a}{{\left(1+{\left(\frac{x}{c}\right)}^{d}\right)}^{f}}\;$$

where *a*, *b*, *c*, *d*, and *f* are constant coefficients, *y* is the net median fluorescence intensity, and *x* is the concentration in pg/ml. In all cases, xPONENT^®^ software for Luminex instrumentation was used for data analysis.

### Data and biostatistical analysis

For values lower than the limit of detection, the same methodology as Dong et al. (23) was used. Values below the limit of detection were substituted by the 10% of the minimum value for each analyte. All concentration values (pg/ml) were converted to log_10_ for further analysis.

**Figure 1:**
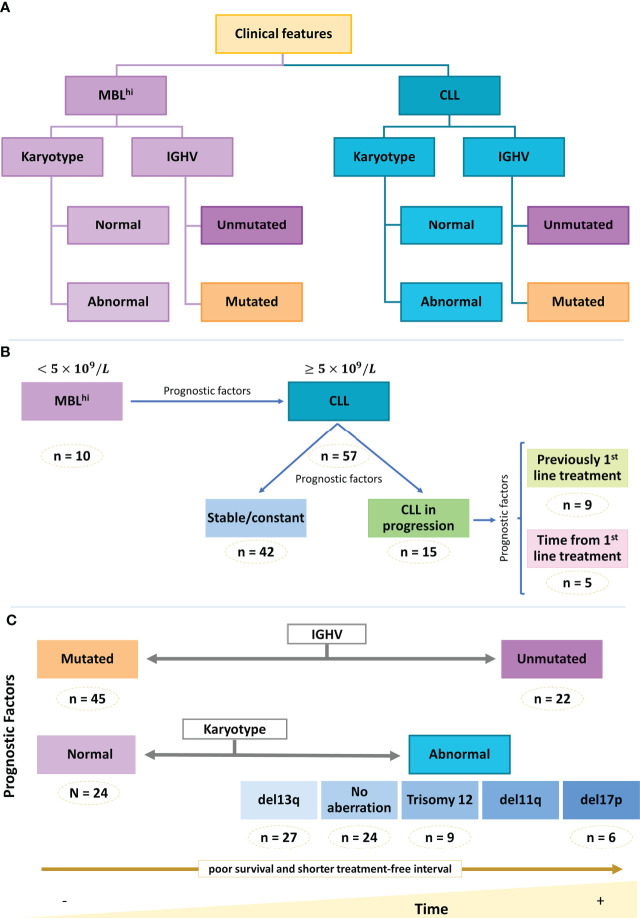
Clinical and biological info of the cohort. Schematic representation of cohort main features. **(A)** Diagnosis. **(B)** Distribution of diagnosis group. **(C)** Main prognostic factors.

For statistical analysis, the samples were pooled according to the clinical characteristics of the patients (**Figure 1**).

The conventional statistical analyses were made with the software R v. 4.1 under RStudio v. 2022.02.0. The normality of the data was studied with the Shapiro–Wilk normality test. Due to the absence of normality of some variables, the correlation between the proteins was carried out using Spearman’s correlation. The homoscedasticity between categories of factor variables was verified with the Fligner–Killeen test and the Bartlett test. According to whether the variables were normal, heteroscedastic, or their possible combinations, the search for significant differences between categories was done with the *t*-test or Wilcoxon–Mann–Whitney test (for two categories) and ANOVA or Kruskal–Wallis (for more than two categories). The *post-hoc* tests used were Tukey, *t*-test, or Wilcoxon depending on the corresponding global test of significant differences. A significance level of 5% was used in all statistical tests (**Supplementary Figure 1**).

In addition, a linear model (based on the limma statistical framework) including the six major covariates (diagnosis, disease status, treatment, IGHV mutational status, age, and sex) was generated to analyze significant differences in protein abundance between the 67 analyzed samples (design matrix: ~ 0 + groups + IGHV mutational status+ age + sex, soluble protein) (24). Pairwise comparisons (MBL vs. CLL; c-CLL vs. p-CLL; c-CLL vs. CLL-PFT; c-CLL vs. CLL-TFT) returned 88, 84, and 64 proteins with significant differences at least in contrast [adjusted *p*-values< 0.1, 0.05, and 0.01, Benjamini–Hochberg (BH) correction for multiple testing].

Representation groups were made with GraphPad Prism Software v. 8.0.2 (GraphPad Software Inc., San Diego, EE.UU.) and Infinicyt™ 2.0.5 (Cytognos SL, Salamanca, Spain). Pathways and functional enrichment were made using Reactome (https://reactome.org) (25).

A quantitative methodology, based on the maximum relevance minimum redundancy (mRMR) scheme (26, 27), was used to rank the proteins by relevance. To make the results more robust, we repeated the mRMR procedure with 1,000 different sets each consisting of a subset with 80% of the available patients selected at random. Subsequently, for each protein, we counted the number of times the proteins belong to the top 5 or top 20, and ranked them using a score based on this information.

Several decision tree models (28, 29) were used to classify (i) monoclonal B-cell lymphocytosis (MBL^hi^) and chronic lymphocytic leukemia stable/constant (c-CLL) or progression (p-CLL), and depending on the treatment line, (ii) the CLL group prior to first-line treatment (CLL-PFT) and another group after first-line treatment (CLL-TFT), and (iii) IGHV mutational status (mutated and unmutated). These decision tree models were carried out based on (i) the top 5 soluble immune factors considered with the mRMR analysis, (ii) proteins with significant differences after conventional statistical analysis, and (iii) the coincident ones obtained with significant differences with the linear model. These results were expressed as confusion matrices for each run, where the out-of-sample error was estimated using k-fold cross-validation (30).

## Results

### Profiling immune soluble factors according to diagnostics stage (MBL^hi^ vs. CLL vs. Stable/constant CLL vs. CLL in progression)

In **Table 1**, the clinical–biological information of the cohort analyzed is described (MBL^hi^ vs. CLL—stable/constant and/or progression). In order to decipher differential protein profiles in all the samples, three groups of soluble proteins were established: (a) group 1: cytokines, chemokines, growth/factors, regulators, and soluble receptors; (b) group 2: soluble immune checkpoints related to T cells; and (c) group 3: soluble immune checkpoints related to NK cells.

Initially, variations in the quantitative values of these soluble immune factors (groups 1, 2, and 3) in serum have been assessed. When analyzing the deviation of the average concentrations for each protein in relation to the average from each group (group 1, 2, or 3) based on diagnostic (MBL^hi^ vs. CLL—stable/constant and/or progression), it has been observed that:

- 33.85% (22/65) of cytokines in group 1 show a deviation from their average concentration with respect to the average levels within this group for the MBL^hi^ and CLL cohort. Of these, 18.46% (12/65) have a higher average distribution compared to all cytokines assessed in the case of MBL^hi^ and 15.38% (10/65) for CLL; in addition, several of these proteins are common to both cases (MBL^hi^ and CLL): IL-16, CXCL5, CXCL13, CCL22/MDC, CCL24/Eotaxin-2, CXCL12/SDF-1α, sIL-2R, sTWEAK, sCD30, sAPRIL, and sTRAIL. On the other side, there is a set of soluble proteins that have lower values compared to the average concentration of soluble proteins studied in both CLL and MBL^hi^ [13.84% (9/65) and 7.69% (5/65), respectively], such as IL-13, IL-1β, IL-7, and IL-8 (**Supplementary Figure 2A**).
- In the case of soluble immune checkpoints assessed in groups 2 and 3, 21.05% (8/38) show a quantitative increase in relation to the average concentration of proteins studied for both groups in the study cohort (MBL^hi^ and CLL) (sTIM3, sCD27, sPD-L2, sBTLA, and sCD276/sB7-H3 or accessory molecules such as sPerforin, sE-Cahderine, and sCD155/sPVR, which are involved in the immunological synapsis). On the other hand, 18.42% (7/38) of soluble immune checkpoints of these groups have a decrease of concentration with respect to the quantitative average for all these molecules of the study (sCD134/sOX40, sPD-L1, sCD47/IAP, sCD48/sBLAST-1, sMICA, sMICB, and sArginase-1) (**Supplementary Figures 2B, C**).

Considering the panel of soluble immune factors detected with significant differences and according to diagnosis, it was further evaluated if there was a trend during the disease progression (comparison of c-CLL and p-CLL). Changes between MBL^hi^, c-CLL, and p-CLL have been observed for the studied protein profile (**Supplementary Figure 3**; **Supplementary Table 2**).

After conventional statistics (Student’s *t*-test or Wilcoxon–Mann–Whitney test for two categories, and ANOVA or Kruskal–Wallis for more than two categories), significant differences were detected for certain proteins such as sTIMD-4, sIDO, sGalectin-9, IL-4, sBTLA, sLAG-3, IFN-γ, and CXCL13 (**Supplementary Figure 4**; **Supplementary Table 3**). Certain proteins presented a significant increase with the progression of the disease (i.e., sGalectin-9, s4-1BB, and sOX40), most of which are involved in cell recruitment (pro-inflammatory response), cell death regulation, cell cycle regulation, and cell chemotaxis (**Supplementary Figure 5**; **Supplementary Table 4**), while other proteins have a significant reduction of concentration (i.e., sCD30, INF-γ, and sIDO) according to disease progression, from MBL^hi^ through c-CLL to p-CLL. These are related to interferon signaling, CD28 co-stimulation, tryptophan catabolism, and immunological synapse (T and B cells) (**Supplementary Figure 6**; **Supplementary Table 5**).

Furthermore, 30.4% (7/23) of the soluble immune factors have significant differences between p-CLL and c-CLL. These are related to cell recruitment and cell death regulation (**Supplementary Figure 7**; **Supplementary Table 4**). In this case, a few of them are highlighted (IL-4, sTIM-3, IL-18, and CXCL9), which directly target T and B cells.

**Figure 2:**
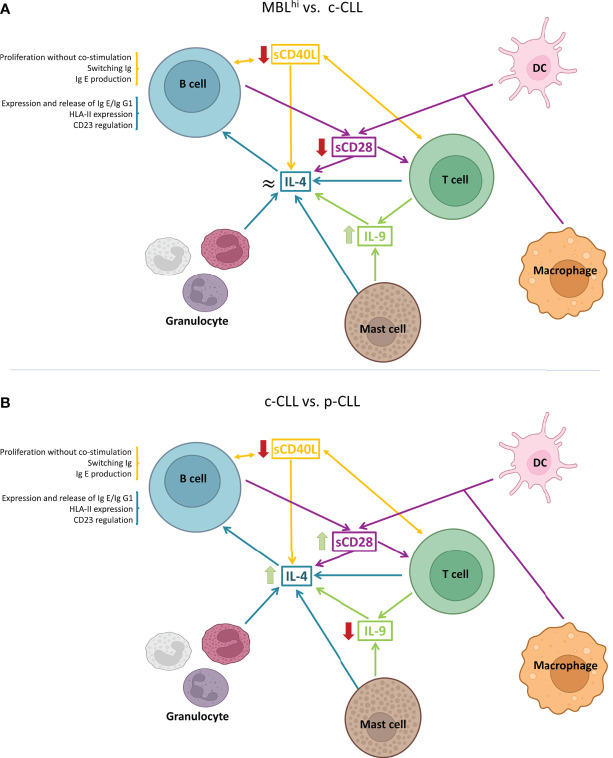
Schematic representation of IL-4-associated differential profiles in TME. **(A)** Cytokine profile observed between MBLhi and c-CLL. **(B)** Cytokine profile between c-CLL and p-CLL. (Created in Biorender.com)

Regarding alterations in IL-4 levels, the detection of differences in p-CLL may be relevant (**Figure 2**). This is because IL-4 production is regulated by immune factors such as CD40L, IL-9, CD28 family, IL-1 family, and IL-13. In addition, it has an important role in TME, as it could be released, for example, by granulocytes (basophils, eosinophils, or mast cells) and T cells. It also has an important role in virus infections and negative regulation of the PI3K/AKT intracellular signaling pathway.

**Figure 3:**
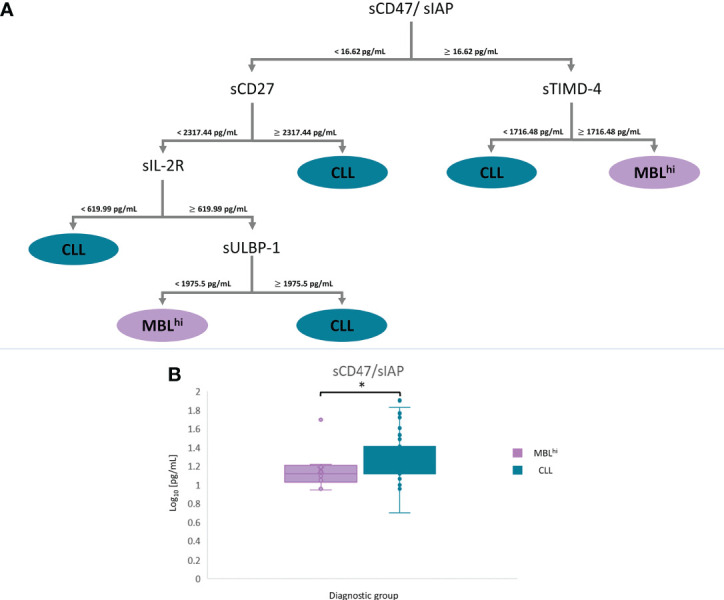
mRMR analysis: MBL^hi^ vs. CLL. **(A)** Distribution and classification of MBL^hi^ and CLL patients from quantitative soluble immune checkpoints and cytokines in serum. **(B)** Boxplot sCD47 in serum between MBL^hi^ and CLL (*p – *value*< 0.05).

**Figure 4:**
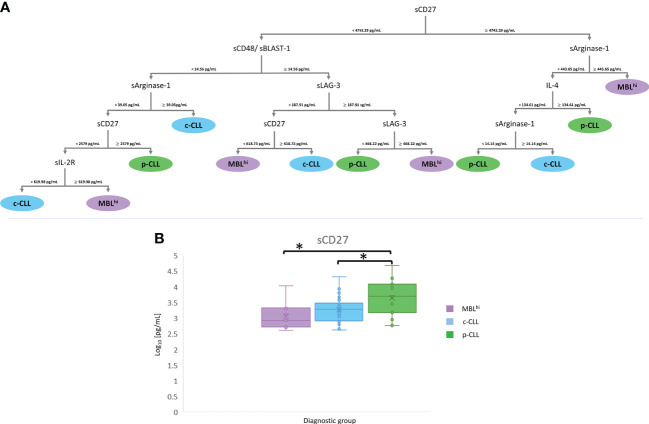
mRMR analysis: MBL^hi^ vs. CLL stages. **(A)** Distribution and classification of MBL^hi^ and c-CLL/p-CLL from quantitative soluble immune checkpoints and cytokines in serum. **(B)** Boxplot sCD27 in serum between MBL^hi^ and CLL stages (*p – *value*< 0.05).

Then, a panel of potential soluble immune factors that are useful in discriminating between diagnosis stage and disease evolution is deciphered and has been obtained from the correlation analysis (**Supplementary Figures 8A,B**; **Supplementary Table 6**), the top five soluble immune factors were obtained by mRMR analysis (**Supplementary Table 7**), and significant proteins from conventional statistics analysis (**Supplementary Table 3**) and proteins with significant differences were detected using the linear model (**Supplementary Table 8**). The set of soluble immune factors that constitute this panel has 71% (CLL) and 90% (MBL^hi^) success in discerning the stage of the disease (**Supplementary Figure 9A**) and 79% (p-CLL), 76% (c-CLL), and 90% (MBL^hi^) regarding the discrimination of the groups according to the disease evolution (**Supplementary Figure 9B**). These consist of the following:

- For *disease stage*: A panel of five soluble immune factors is obtained, which is feasible to discriminate between MBL^hi^ and CLL: sCD47 (cutoff values<16.62 pg/ml to select the sCD27 branch and ≥16.62 pg/ml to select the sTIMD-4 branch), sTIMD-4 (values<1,716.48 pg/ml for CLL and ≥1,716.48 pg/ml for MBL^hi^), sCD27 (≥2,317.44 pg/ml for CLL), sIL-2R (<619.99 pg/ml for CLL), and sULBP-1 (<1,975.5 pg/ml for MBL^hi^ and ≥1,975.5 pg/ml for CLL) (**Figure 3** and **Table 3**).
- For *disease evolution*: A panel of six soluble immune factors is obtained, which is feasible to distinguish between MBL^hi^ and c-CLL, and between MBL^hi^ and p-CLL: sCD48 (<14.56 pg/ml to select the sArginase-1 branch and ≥14.56 pg/ml to select the sLAG-3 branch), sCD27 (<618.73 pg/ml for MBL^hi^ and ≥618.73 pg/ml for c-CLL or ≥2,579 pg/ml for p-CLL), sArginase-1 (≥39.05 pg/ml for c-CLL, ≥443.65 pg/ml for MBL^hi^,<14.14 pg/ml for p-CLL, and ≥14.14 pg/ml for c-CLL), sLAG-3 (<468.22 pg/ml for p-CLL and ≥468.22 pg/ml for MBL^hi^), IL-4 (≥134.61 pg/ml for p-CLL), and sIL-2R (<619.98 pg/ml for c-CLL and ≥619.98 pg/ml for MBL^hi^) to discriminate disease evolution (**Figure 4** and **Table 3**).

**Table 3:** List of relevant soluble immune factors according to stage and evolution disease.

Comparison	Protein	UniProt ID	Target	Statistical analysis
MBL^hi^ vs. CLL	sCD47/sIAP	Q08722	Immune Checkpoint LT	mRMR/conventional statistics/limma-package
	sCD27	P26842	Immune Checkpoint LT	mRMR/conventional statistics/limma-package
	TIMD-4	Q96H15	Immune Checkpoint LT	mRMR/conventional statistics/-
	sIL-2R	P31785	Soluble receptor	mRMR/conventional statistics/limma-package
	sULBP1	Q9BZM6	Immune Checkpoint NK	mRMR/-/limma-package
MBL^hi^ vs. c-CLL vs. p-CLL	sCD48/sBLAST-1	P09326	Immune Checkpoint LT	mRMR/-/-
	sCD27	P26842	Immune Checkpoint LT	-/conventional statistics/-
	sArginase-1	P05089	Immune Checkpoint NK	mRMR/-/-
	sLAG-3	P18627	Immune Checkpoint LT	mRMR/conventional statistics/-
	IL-4	P05112	Cytokine	mRMR/conventional statistics/limma-package
	sIL-2R	P31785	Soluble receptor	-/conventional statistics/limma-package
MBL^hi^ vs. C-CLL vs. CLL-PFT vs. CLL-TFT	sIL-2R	P31785	Soluble receptor	mRMR/conventional statistics/limma-package
	sTIMD-4	Q96H15	Immune Checkpoint LT	mRMR/conventional statistics/-
	sSiglec-9	Q9Y336	Immune Checkpoint NK	mRMR/-/limma-package
	INF-γ	P01579	Cytokine	mRMR/conventional statistics/-
	sPD-L1	Q9NZQ7	Immune Checkpoint LT	mRMR/-/limma-package
	sCD48/sBLAST-1	P09326	Immune Checkpoint LT	mRMR/-/-
	sLAG-3	P18627	Immune Checkpoint LT	mRMR/conventional statistics/limma-package
Mutated IGHV vs. Unmutated IGHV	CXCL10/IP-10	P02778	Chemokine	-/conventional statistics/-
	sCD134/sOX40	P23510	Immune Checkpoint LT	mRMR/-/-
	sULBP1	Q9BZM6	Immune Checkpoint NK	mRMR/-/-
	sLAG-3	P18627	Immune Checkpoint LT	mRMR/conventional statistics/-

mRMR, maximum relevance minimum redundancy.

### Profiling immune soluble factors according to response to therapy

The therapeutic algorithm used in CLL includes several targeted oncotherapies according to multiple prognostic factors and treatment resistances. In this study, potential biomarkers are explored to determine the differences in profiles of soluble immune factors before/after treatment at the different disease stages and during disease evolution.

At first glance, approximately 20% (13/65) of the soluble immune factors in group 1 show a deviation with respect to the average of global group 1. Of these, 18.46% (12/65) of soluble immune factors have higher average concentrations (IL-16, CXCL13, CCL22/MDC, CCL24/Eotaxin-2, CXCL5, CXCL12/SDF-1α, sIL-2R, sTRAIL, sCD30, sTWEAK, sAPRIL, and CCL2/MCP-1), while 16.92% (11/65) of soluble immune factors have lower average concentrations (IL-10, IL-1β, IL-7, IL-8, IL-13, TNF-α, IL-1α, IL-15, CX3CL1/Fractalkine, β-NGF, and sCD40L) (**Supplementary Figure 10A**).

Regarding soluble immune checkpoints (groups 2 and 3), 23.68% (9/38) of them show an increase in regard to the average concentration for each group [sPerforin, sCD276/sB7-H3 and sPD-L2, several receptors (sCD27, sBTLA, sTIM3, and s4-1BB/sCD137), and adhesion proteins (sE-Cadherin and sCD155/sPVR)], while on the other hand, 21.05% (8/38) of soluble immune checkpoints have downward deviations from average concentrations for all these groups (sCD134/sOX40, sPD-L1, sCD48/sBLAST-1, sCD47/sIAP, sULBP-3, sArginase-1, and, related to antigen presentation, sMICA and sMICB) (**Supplementary Figures 10B,C**).

To establish a pattern of soluble immune factors useful to discriminate between diagnostic stage and response to treatment (before/after therapy), firstly, the profile tendency of the soluble immune factors is studied. An increase in them is observed in CLL-PFT (**Supplementary Figure 11**), where this profile is related to cellular senescence, post-translational protein phosphorylation, PI3K/AKT intracellular signaling, and virus infection, among others. However, from the correlation analysis with disease progression, a profile with indirect correlation is observed, since the quantitative levels are lower after treatment. When performing a functional analysis, it is observed that among the cell signaling pathways involved are TLR signaling cascade, response to infection, regulation of gene expression by hypoxia-inducible factor, apoptotic cleavage of cell adhesion proteins, extracellular matrix organization, cell–cell communication, signaling transduction, and transcription regulation of pluripotent stem cells (**Supplementary Table 9**).

Some of the significant soluble immune factors are sIL-2R, CXCL9/MIG, sTIMD-4 for MBL^hi^ vs. CLL-PFT, sIDO, IL-18 and sCD30 for c-CLL vs. CLL-PFT or sLAG-3, and IFN-γ for c-CLL vs. CLL-TFT. All significant soluble immune factors for these comparisons are reported in **Supplementary Table 2**, and their trend is shown in **Supplementary Figure 12**. After functional enrichment, most of the soluble immune factors reveal signaling pathways related to co-stimulation mediated by the CD28 family in the immune system and immune-related signaling. For the comparison of c-CLL vs. CLL-PFT, differential signaling pathways are related to metabolism of amino acids and the programmed cell death (**Supplementary Table 10**).

**Figure 5:**
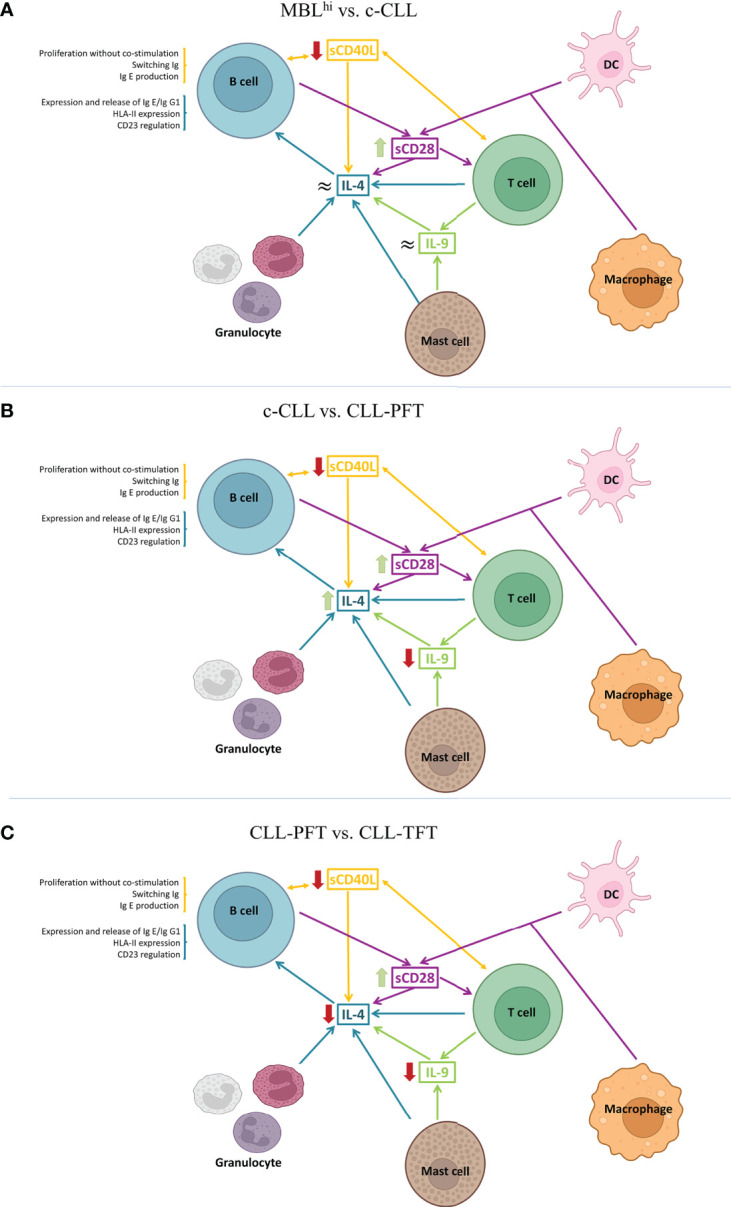
Schematic models of the TME from the role of the observed differential serum profiles. **(A)** MBLhi vs. c-CLL (stable and progression). **(B)** c-CLL vs. CLL-PFT. **(C)** CLL-PFT vs. CLL-TFT. (Created in Biorender.com)

As described above on the IL-4 quantitative levels, this increases with the evolution of the disease, but it is decreased in CLL-PFT. Considering a detailed study of soluble immune factors involved in the regulation of IL-4 production, these show that a decrease in sCD40L and sCD28 is associated with an increase in disease progression (**Figure 5**).

**Figure 6:**
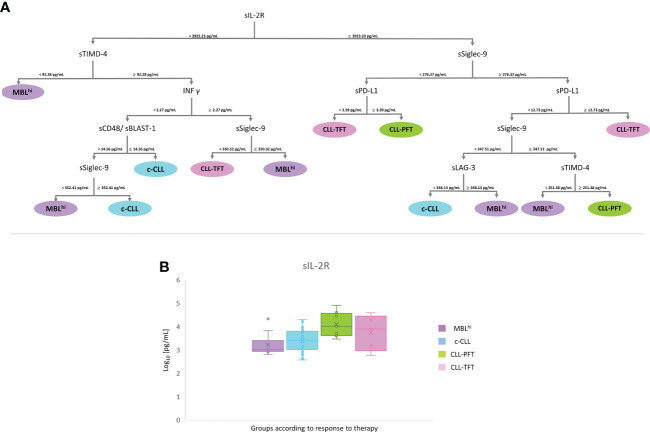
mRMR analysis according to therapy. **(A)** Distribution and classification from differential profiles of soluble receptors and immune checkpoints. **(B)** Boxplot sIL-2R in serum across the analyzed cohort.

Similarly, from the correlation analysis (**Supplementary Figure 8C**; **Supplementary Tables 3**, **6**–**8**), it is feasible to establish a pattern of soluble immune factor to successfully discriminate between groups according to treatment response, which yields the following rates: 48% (c-CLL), 93% (MBL^hi^), 95% (CLL-PFT), and 100% (CLL-TFT) (**Supplementary Figure 9C**). Thus, a panel containing sIL-2R (<3,923.23 pg/ml to select the sTIMD-4 branch and ≥3,923.23 pg/ml to select the sSiglec-9 branch), sTIMD-4 (<92.28 pg/ml for MBL^hi^ or<251.38 pg/ml for MBL^hi^ and ≥251.38 pg/ml for CLL-PFT), sSiglec-9 (<330.32 pg/ml for CLL-TFT and ≥330.32 pg/ml for MBL^hi^ or<352.41 pg/ml for MBL^hi^ and ≥352.41 pg/ml for c-CLL), INF-γ (<2.27 pg/ml to select the sCD48 branch and ≥2.27 pg/ml to select the sSiglec-9 branch), sPD-L1 (<3.39 pg/ml for CLL-TFT and ≥3.39 pg/ml for CLL-PFT or ≥12.73 pg/ml for CLL-TFT), sCD48 (≥14.56 pg/ml for c-CLL), and sLAG-3 (<348.13 pg/ml for c-CLL and ≥348.13 pg/ml for MBL^hi^) could distinguish between diagnostic stage and therapeutic response (**Figure 6** and **Table 3**).

### Profiling soluble immune factors according to immunoglobulin heavy chain variable mutational status

In this study, the profiles of soluble immune factors based on immunoglobulin heavy chain variable (IGHV) mutational status are also analyzed due to the critical role as a prognostic factor in CLL.

In **Supplementary Figure 13**, the general distribution of soluble immune factors for both IGHV mutational status (unmutated vs. mutated) is depicted. In group 1 of soluble immune factors, 16.90% (11/65) show higher levels with respect to the global average (IL-16, CXCL5, CCL24/Eotaxin-2, CXCL12/SDF-1α, sIL-2R, sTWEAK, sCD30, and sAPRIL), while 15.38% (10/65) of soluble immune factors show decreased levels (IL-1α, IL-7, IL-13, IL-15, IL-10, TNF-α, IL-1β, IL-8, CXC3CL1/fractalkine, and β-NGF) (**Supplementary Figure 13A**).

With regard to soluble immune checkpoints (T cells or NK cells), 18.42% (7/38) of them, included in both groups, have an increased average concentration concerning the total average for each group (sBTLA, sCD276/sB7-H3, sTIM-3, sCD27, sPerforin, sE-Cadherin, and sCD155/sPVR). Likewise, a reduction in quantitative levels is also observed for 15.79% (6/38) of the soluble immune checkpoints included in these groups (sPD-L1, sCD47/sIAP, sCD134/sOX40, sMICA, and sMICB) (**Supplementary Figures 13B, C**).

Most of the studied soluble immune factors [83.5% (86/103)] show higher levels for unmutated IGHV patients than mutated IGHV patients. These results are to be expected due to the poor clinical prognosis of unmutated IGHV patients (**Supplementary Figure 14**). From the functional point of view, these soluble immune factors are related to CD28 co-stimulation, regulation of TLR by endogenous ligand, infectious disease, constitutive signaling by aberrant PI3K, adherens junction interactions, cellular response to stress, regulated necrosis, and pyroptosis (**Supplementary Table 11**). Although only 9.7% (10/103) of soluble immune factors are significantly increased (**Supplementary Figure 13**). The last mentioned factors are related to VEGF signaling, immunoregulatory interactions between lymphoid and non-lymphoid cells, and transcriptional regulation, among others (**Supplementary Table 11**).

In this comparison, CXCL11/I-TAC, CXCL10/IP-10, sHVEM, and sLAG-3 are found to have a significant profile for IGHV mutational status (**Supplementary Table 3**), which is related to antigen presentation and cytokine signaling cascades (i.e., IL-10 signaling, TNFR2 non-canonical NF-κB pathway, and interferon signaling) (**Supplementary Table 12**).

**Figure 7:**
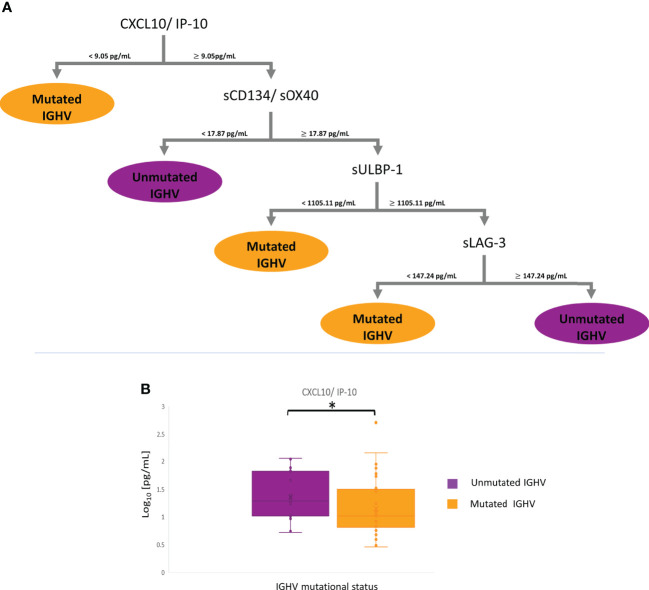
mRMR analysis according to IGHV status. **(A)** Distribution and classification from differential profiles of soluble immune checkpoints correlated with IGHV mutational status. **(B)** Boxplot CXCL10/IP-10 in serum between groups (*p – *value*< 0.05).

Bearing in mind the prognostic value of the IGHV mutational status in CLL, it is very interesting to identify a profile of soluble immune checkpoints with a strong correlation. In this way, a panel of four proteins is feasible to discriminate IGHV mutational status: CXCL10/IP-10 (<9.05 pg/ml for mutated IGHV), sCD134/sOX40 (<17.87 pg/ml for unmutated IGHV), sULBP-1 (<1,105.11 pg/ml for mutated IGHV), and sLAG-3(<147.24 pg/ml for mutated IGHV and ≥147.24 pg/ml for unmutated IGHV) (**Figure 7** and **Table 3**). This panel has a high discrimination capacity between groups (70% for mutated IGHV and 77% for unmutated IGHV) (**Supplementary Figure 8D**, **Supplementary Figure 9D**, and **Supplementary Tables 3**, **6**–**8**), and all of them are involved in interactions with T cells and the mechanism of antigen presentation.

## Discussion

In this study, serum quantitative levels of 103 soluble immune factors have been analyzed in MBL^hi^/CLL patients. The characterization of significant profiles of soluble immune factors reveals patterns and trends that might be useful as potential biomarkers to distinguish between diagnostic stage, disease evolution, and prognostics factors (such as IGHV mutational status). Briefly, it has been observed that soluble immune factors show an increment according to diagnostic stage (MBL^hi^ vs. CLL), disease evolution (c-CLL and p-CLL), and therapy response (c-CLL and p-CLL) (CLL-PFT vs. CLL-TFT). On the other hand, after treatment, a common reduction in soluble immune factors is observed. Despite these patterns, an exception has been determined as sLAG-3, sPD-L1, and INF-γ have reduced levels at the early stage of the disease. As regards prognostic factors, unmutated IGHV patients display lower levels of soluble immune factors in comparison with mutated IGHV, except for sCD47/sIAP and sTIMD-4, which maintain their levels, and sArginase-1, which shows increased levels.

**Figure 8:**
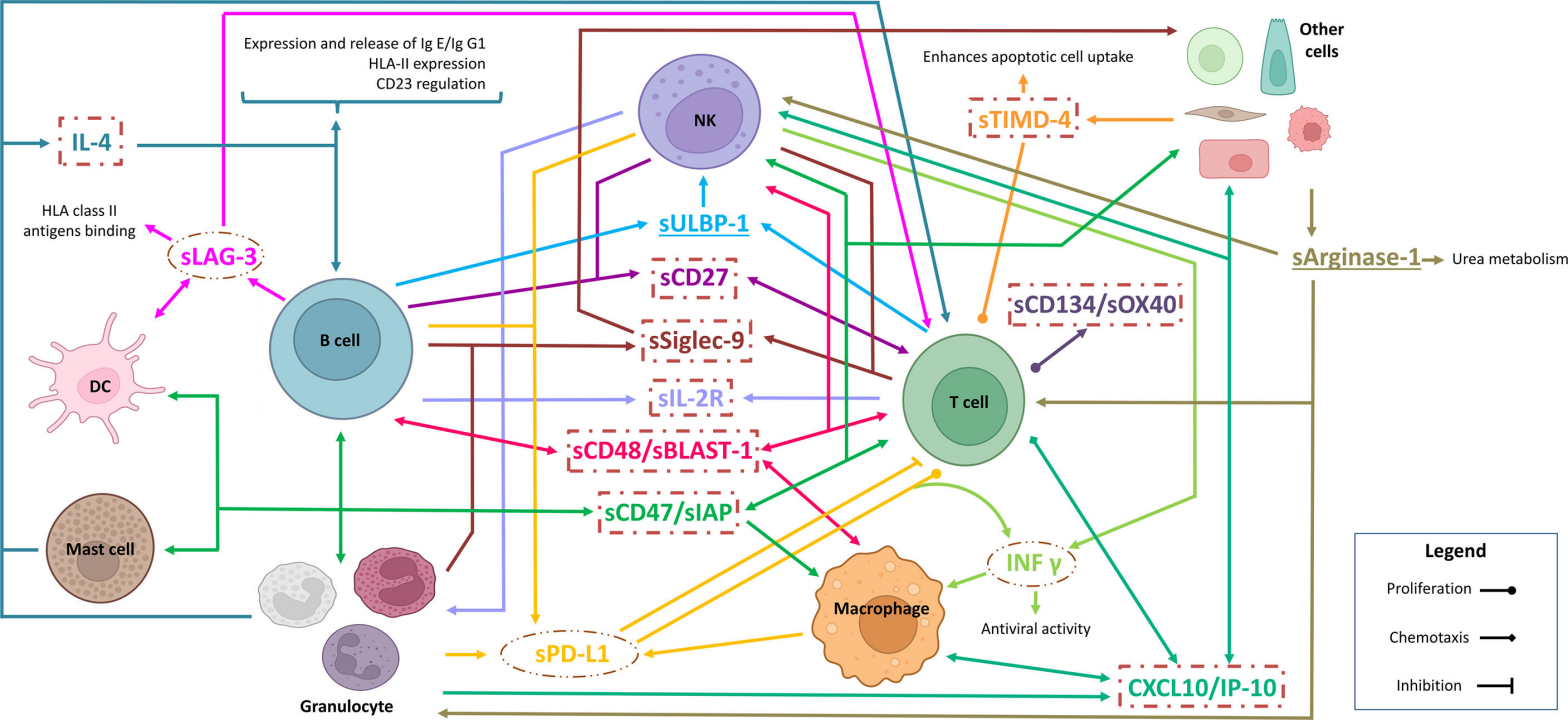
Model of the role of soluble immune checkpoints in the TME in the studied cohort. Inside circles, protein profiling decreased levels according to disease evolution. Inside squares, protein profile correlated with mutated IGHV. Underline, protein profiles with fluctuations over disease evolution and IGHV mutational status. (Created in Biorender.com)

The significant profiles of soluble immune factors are directly related to the microenvironment where T cells, NK cells, macrophages, and granulocytes are involved (**Figure 8**). As a proof, IL-4 is increased according to disease evolution. IL-4 is a cytokine that polarizes T-lymphocyte differentiation towards Th2 cells (1) and macrophages towards pro-tumoral (M2) phenotypes, leading to tumor escape caused by an unbalanced immune response. Previously, it has been reported that IL-4 is produced by B-CLL cell (2) and in T-cell cultures isolated from CLL patients (31). This general immunosuppression increased the survival of B-CLL cells and the modulation of the therapeutic response of CLL patients (1, 10, 32). This is compatible with the current study, since the maintenance or increment in IL-4 serum concentrations could be driven by the polarization of the microenvironment, skewing the action of the immune system towards a pro-tumor microenvironment, which would be restored after targeted therapy.

Bearing in mind the polarization of Th2 cells in the microenvironment and the increased presence of M2 macrophages (releasing IL-4 into the microenvironment), Arginase-1 is also expected to be involved. Arginase-1 production is stimulated by IL-4 production, thus supporting the effect of Th2 cells (33). In addition, Arginase-1 could also be released into the microenvironment by myeloid cells; thus, it contributes to the suppression of T-cell functions and inhibits NK proliferation. Alterations in arginase metabolism have also been observed in tumors, which might affect cell proliferation. On the other hand, tumor cells have been shown to exhibit an increase in arginase catabolism that leads to the suppression of CD8^+^ T cells and stabilizes Treg T cells (34, 35). In this study, it is observed that sArginase-1 increases its concentration according to disease progression. In particular, CLL-PFT patients display a lower level compared to patients with c-CLL and show an increase when compared with CLL-TFT. This suggests that the catabolism of Arginase-1 is increased in B-CLL cells, generating a protective environment for these leukemic cells that allows their further proliferation and, consequently, treatment resistance.

Also, the current study reveals the critical role of T cells in disease progression and evolution. Significant alterations in soluble immune factors related to T-cell functions are as follows: (i) sULBP-1 is relevant in the inhibition of cytokine release, co-stimulation, and cytotoxicity of T cells (6, 36). (ii) sSiglec-9, which could bind to its ligands, promotes the block of antitumoral activity of NK and T cells (37). (iii) sLAG-3 inhibits the co-receptor of HLA-II that downregulates the activation of T and NK cells to alert the exhaustion status of T and NK cells (12, 38, 39). Sordo-Bahamonde et al. in 2021 (11) observed that LAG-3 has a negative impact on the clinical outcomes of CLL patients, affecting T-cell proliferation, cytokine production, and cytolytic activity, while promoting immunosuppression by Tregs’ T-cell action. Moreover, LAG-3 dysregulation in CLL patients correlates with disease progression. (iv) PD-L1 binding to its receptor PD-1 inhibits T cells (40); increased soluble levels in plasma are associated with poor prognosis, shorter survival, and resistance to immunotherapy in different several cancers (41). In summary, a dysfunctional immune response is expected because of the reduction in antigen presentation by tumor cells and the decrease in the number of T and NK cells, which result in disease progression and no change in the serum levels of sLAG-3 and sPD-L1 after treatment, thus generating treatment resistance.

Interestingly, the sCD48 profile is correlated to the progressive increment with disease progression and evolution. CD48 is a membrane protein that stimulates the cytotoxic activity of CD8^+^ T cells and NK cells (42). This might be related to the response of CLL patients when they are exposed to recurrent viral and bacterial infections. Previously, it has been reported that infected patients show high CD48 expression (43), which is in contrast with what is observed in other hematological malignancies, where downregulation of CD48 has been determined (44). In addition, the presence of elevated levels of sCD48 may be involved with an overstimulation of NK and CD8^+^ T cells, which could block the activity of these cells due to depletion (42). Similarly, it is expected for sCD47. CD47 is a transmembrane protein that plays an important role in migration, phagocytosis, apoptosis, and immune homeostasis because it is responsible for the immune escape control. In normal tissues, there is a constant balance between inhibition/activation of phagocytosis that is disproportionate in malignant cells, mainly because of the upregulation of CD47. In addition, CD47 interacts with signal regulatory protein α (SIRPα) expressed in myeloid cells (monocytes, macrophages, and DCs), blocking the migration and phagocytosis process of these cell types (12, 16, 17, 40).

Finally, from the analysis of soluble immune checkpoint according to the IGHV mutational status, it is revealed that patients with unmutated IGHV are characterized by (i) the proliferation of compromised T cells [sLAG-3 (11, 12), INF-γ (44), sOX40/sCD134 (45), and sCD27 (46)]; (ii) inefficient antigen presentation by APCs [downregulation of HLA-II molecule expression by B-CLL cells, blockade of the HLA-II molecule by sLAG-3 (11, 12), upregulation of IL-4 (1, 10), and blocking of antigenic presentation by DCs by sTIMD-4 (47)]; (iii) the inhibition of CD8^+^ T cells that co-exists with the recruitment and activation of NK [CXCL10 (19), sULBP-1 (6, 36), and sCD48/sBLAST-1 (44)]; and (iv) the inhibition of phagocytosis in macrophages by the binding of sCD47 to SIRPα (12, 16, 17, 40), which, in combination with inefficient antigenic presentation, promotes B-CLL cell survival. Regarding sArginase-1, mutated IGHV patients display higher values than unmutated IGHV patients, which is in contrast with the previously reported level of sArginase-1 in TME, as it is involved in the stabilization of Treg T cells, generating protection for B-CLL cells and suppressing CD8^+^ T cells (34).

## Conclusions

In conclusion, the systematic analysis of soluble immune factors provides a new perspective on the understanding of the TME of B-CLL cells, suggesting that T cells have an important role in this process of immune suppression and dysfunction. This is because most of the soluble immune checkpoints and immune factors that presented significant differences are related to the following:

- Antigenic recognition by T lymphocytes, and co-stimulation, differentiation, proliferation, and selection of circulating tumor-associated monocytes.
- Breakdown of the cellular balance due to the increase in Th2 CD4^+^ T cells, Treg lymphocytes, and type 2 macrophages (M2), which is related to the polarization of the microenvironment. Consequently, an inhibition of the immune system and the presence of an inflamed TME leading to tumor escape are observed.

In this study, four different quantitative profiles of soluble immune factors are proposed. Furthermore, this study paves the way for soluble immune factors as potential and useful biomarkers for this pathology, which could influence disease progression.

## Data availability statement

The datasets presented in this study can be found in online repositories. The names of the repository/repositories and accession number(s) can be found in the article/**Supplementary Material**.

## Ethics statement

Informed consent was given by each individual before entering the study and approved by the local ethics committee of the University Hospital of Salamanca (HUS, Salamanca, Spain).

## Author contributions

Conceptualization: AL-V, MA, AN-B, MG, MF. Sample acquisition and informed consents: AN-B, MG, MA. Statistical Data: AL-V, LD-M, JS-S. Bioinformatic analysis; AL-V, CP, QL, MF. Bioinformatic resources; CP, QL, JDLR. Resources: CA-H, PJ-V, Á-PH, MG-V, AO. Writing-original draft preparation: AL-V, MF. Supervision: MF, MG, AO,CP, QL, JS-S, RG, JD. Funding acquisition: MF. All authors contributed to the article and approved the submitted version.

## Funding

We gratefully acknowledge Fondos FEDER (EU) and Junta Castilla-León (grant SA198A12-2 and COVID-19 grant COV20EDU/00187), Fundación Solórzano FS/38-2017. The Proteomics Unit belongs to ProteoRed, PRB3-ISCIII, supported by grant PT17/0019/0023, of the PE I + D + I 2017-2020, funded by ISCIII and FEDER. AL-V is supported by VIII Centenario-USAL PhD Program, and PJ-V is supported by the JCYL PhD Program “JCYL Nos Impulsa” and scholarship JCYL-EDU/601/2020. This research work was performed in the framework of the Nanomedicine CSIC HUB (ref. 202180E048). AL-V, CA-H, PJ-V, MG-V, ÁP-H, and MF are part of the CSIC’s Global Health Platform (PTI SaludGlobal). CA-H is supported by Instituto de Investigación Biomédica de Salamanca, IBSAL (Programa Puente Contratos Predoctorales-2021).

## Acknowledgments

We thank the financial support from the Spanish Health Institute Carlos III (ISCIII) (grants FIS PI21/01545, FIS PI17/01930, and CB16/12/00400).

## Conflict of interest

The authors declare that the research was conducted in the absence of any commercial or financial relationships that could be construed as a potential conflict of interest.

## Publisher’s note

All claims expressed in this article are solely those of the authors and do not necessarily represent those of their affiliated organizations, or those of the publisher, the editors and the reviewers. Any product that may be evaluated in this article, or claim that may be made by its manufacturer, is not guaranteed or endorsed by the publisher.
