# Supplementary material for: Unravelling soluble immune checkpoints in chronic lymphocytic leukemia: Physiological immunomodulators or immune dysfunction
Source: Front Immunol. 2022 Sep 28;13:965905. doi: 10.3389/fimmu.2022.965905 (PMC9554405; doi:10.3389/fimmu.2022.965905)
Supplement: Supplementary file 1 [file DataSheet_1.pdf]

Supplementary Material

1 Supplementary Tables

Supplementary Table 1: Clinical data of the study cohort.

Supplementary Table 2: Functional enrichment by trend of soluble proteins for Diagnostic group study.

Supplementary Table 3: Summary of significant soluble proteins for each study after classical statistical analysis.

Supplementary Table 4: Functional enrichment of significant soluble proteins for comparison Monoclonal B-cell lymphocytosis (MBL^{hi}) vs. Chronic Lymphocytic Leukemia (CLL) and CLL in progression (p-CLL) vs. Stable/constant CLL (c-CLL) vs. MBL^{hi} and combinations.

Supplementary Table 5: Functional enrichment of significant soluble proteins according to trend for Diagnostic group study.

Supplementary Table 6: Protein correlation.

Supplementary Table 7: Top 20/30 proteins after random forest analyses.

Supplementary Table 8: Summary of significant soluble proteins for each study after linear model.

Supplementary Table 9: Functional enrichment by trend of soluble proteins for Diagnostic group and treatment line.

Supplementary Table 10: Functional enrichment of significant soluble proteins for all combination combinations between Monoclonal B-cell lymphocytosis (MBL^{hi}), Stable/constant Chronic Lymphocytic Leukemia (c-CLL), CLL in progression previously to 1st line treatment (CLL-PFT) and CLL in progression to time from 1st line treatment (CLL-TFT).

Supplementary Table 11: Functional enrichment by trend of soluble proteins for Immunoglobulin Heavy chain Variable (IGHV) gene status study.

Supplementary Table 12: Functional enrichment of significant soluble proteins for Immunoglobulin Heavy chain Variable (IGHV) gene status study.

2. Supplementary Figures

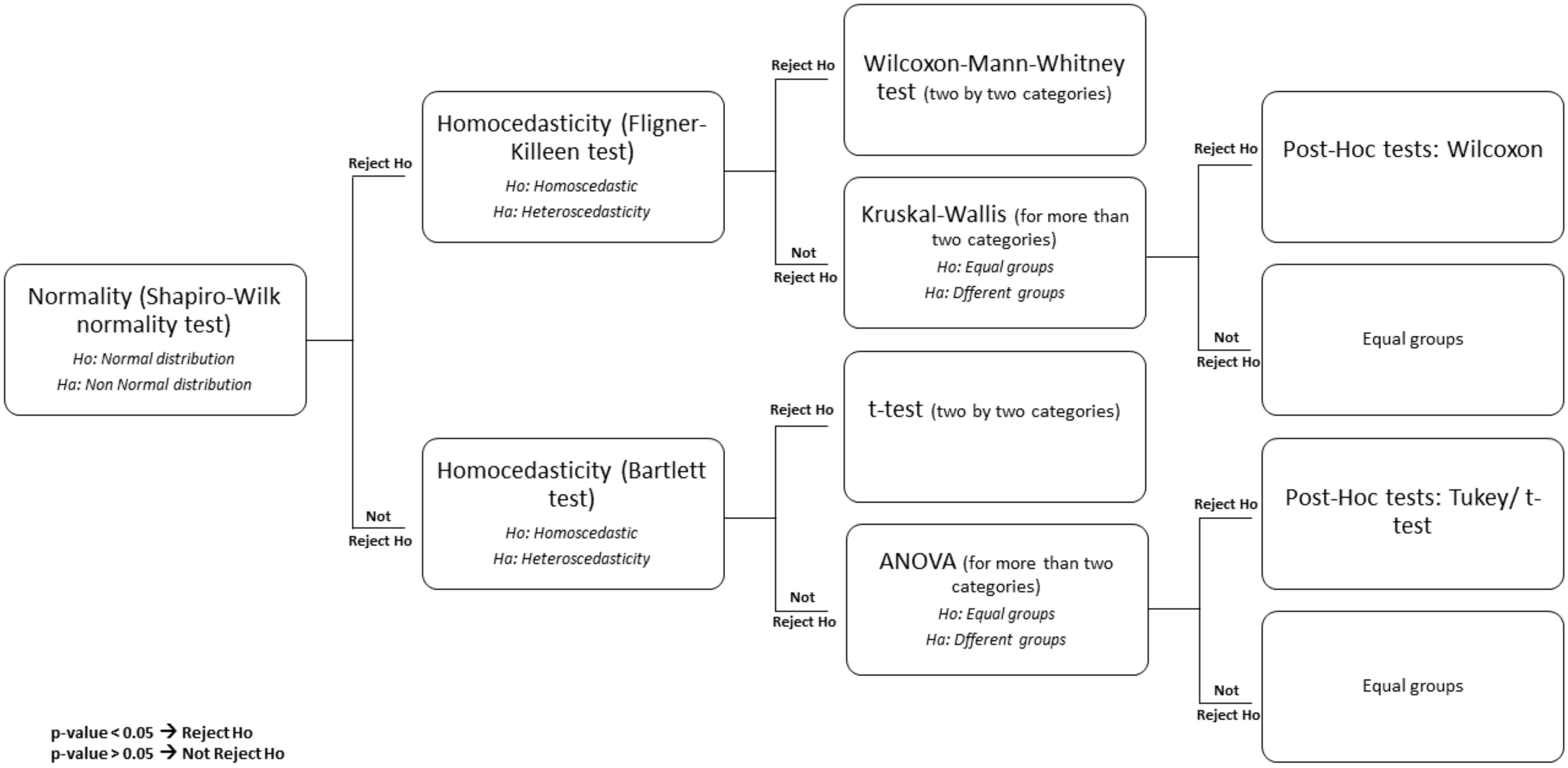

Supplementary Figure 1: Workflow used for conventional statistic analyses. Scheme of the steps taken from a statistical point of view.

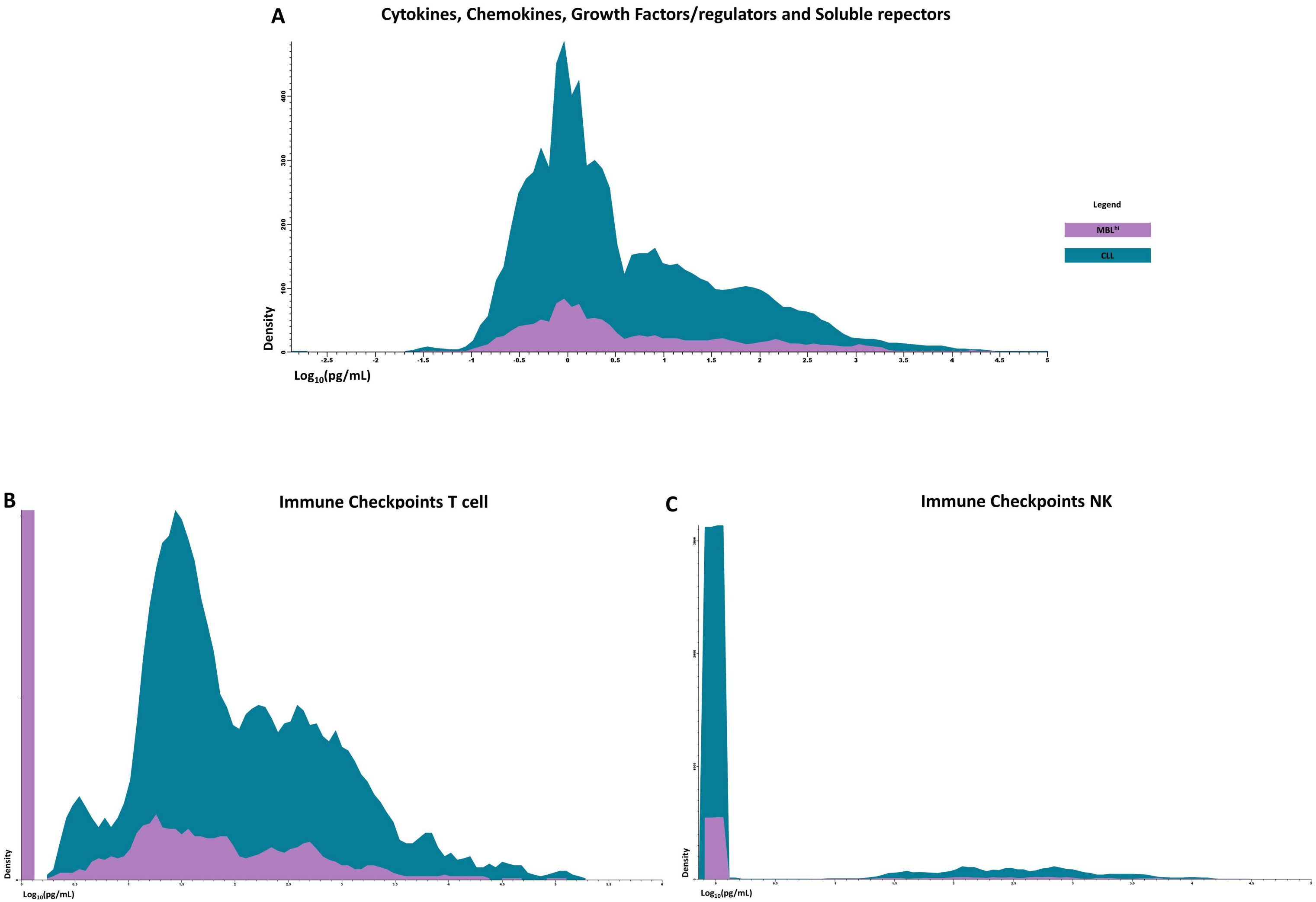

Supplementary Figure 2: Density profiles of soluble proteins according clinical main groups. Panel A.-Group 1: Cytokines, chemokines, growth factors/regulators and soluble receptors. Panel B.-Group 2. Soluble immune checkpoints related to T cells. Panel C.-Group 3.-Soluble immune checkpoints related to NK cells.

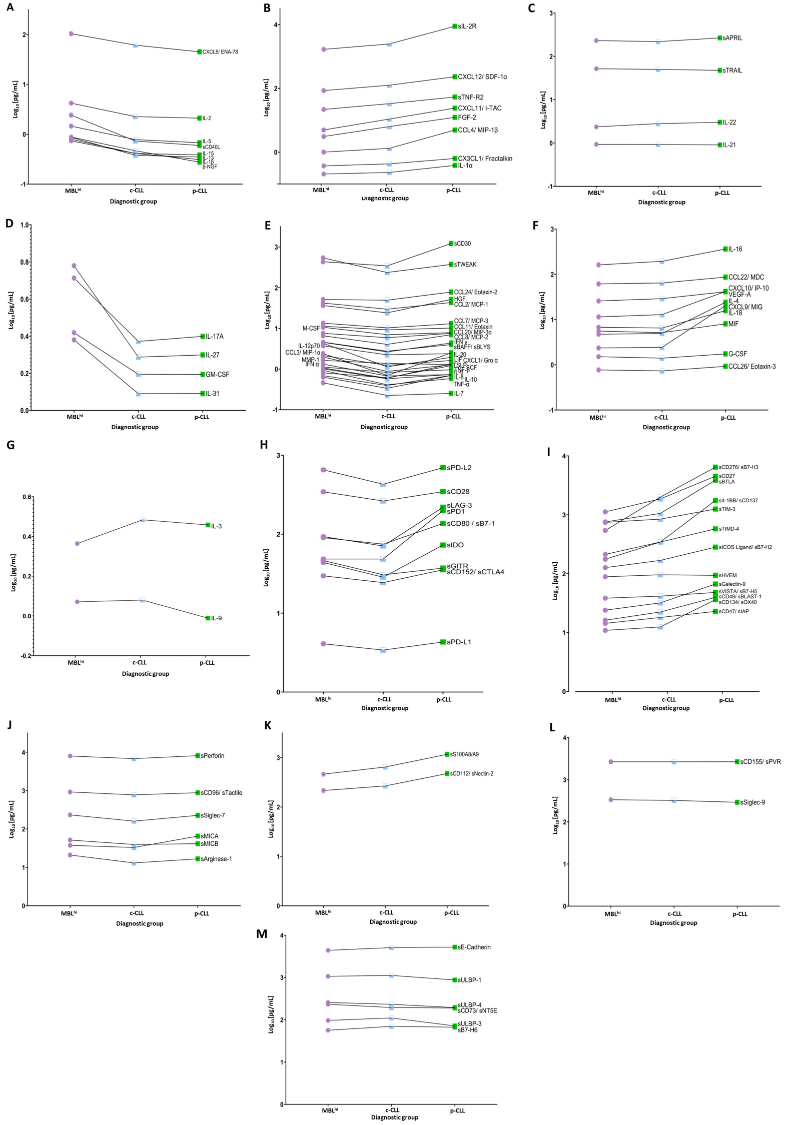

Supplementary Figure 3: Quantitative profiles of soluble proteins studied in multiple comparisons across main clinical features and disease evolution. Panel A.- Decreased profile cytokines, chemokines, growth factors/regulators and soluble receptors. Panel B.- Increased concentration for cytokines, chemokines, growth factors/regulators and soluble receptors. Panel C.- No alteration in quantitative levels for cytokines, chemokines, growth factors/regulators and soluble receptors. Panel D.- Protein profiles (cytokines, chemokines, growth factors/regulator and soluble receptors) with a decreased levels according disease evolution. Panel E.- Protein profiles (cytokines, chemokines, growth factors/regulators and soluble receptors) with low quantitative level in c-CLL. Panel F.- Proteins profiles (with high quantitative level on p-CLL. Panel G.- Protein profile (cytokines, chemokines, growth factors/regulators and soluble receptors) with increment in CLL. Panel H.- Soluble immune checkpoints of T cells with higher levels on CLL in progression. Panel I.- Soluble immune checkpoints of T cells with increased levels according disease evolution. Panel J.- Increased quantitative level for Immune checkpoints of Natural killer cell. K) Increased concentration for Immune checkpoints of Natural killer cell. L) Similar trend of concentration for Immune checkpoints of Natural killer cell. M) Trend combination of concentration for Immune checkpoints of Natural killer cell.

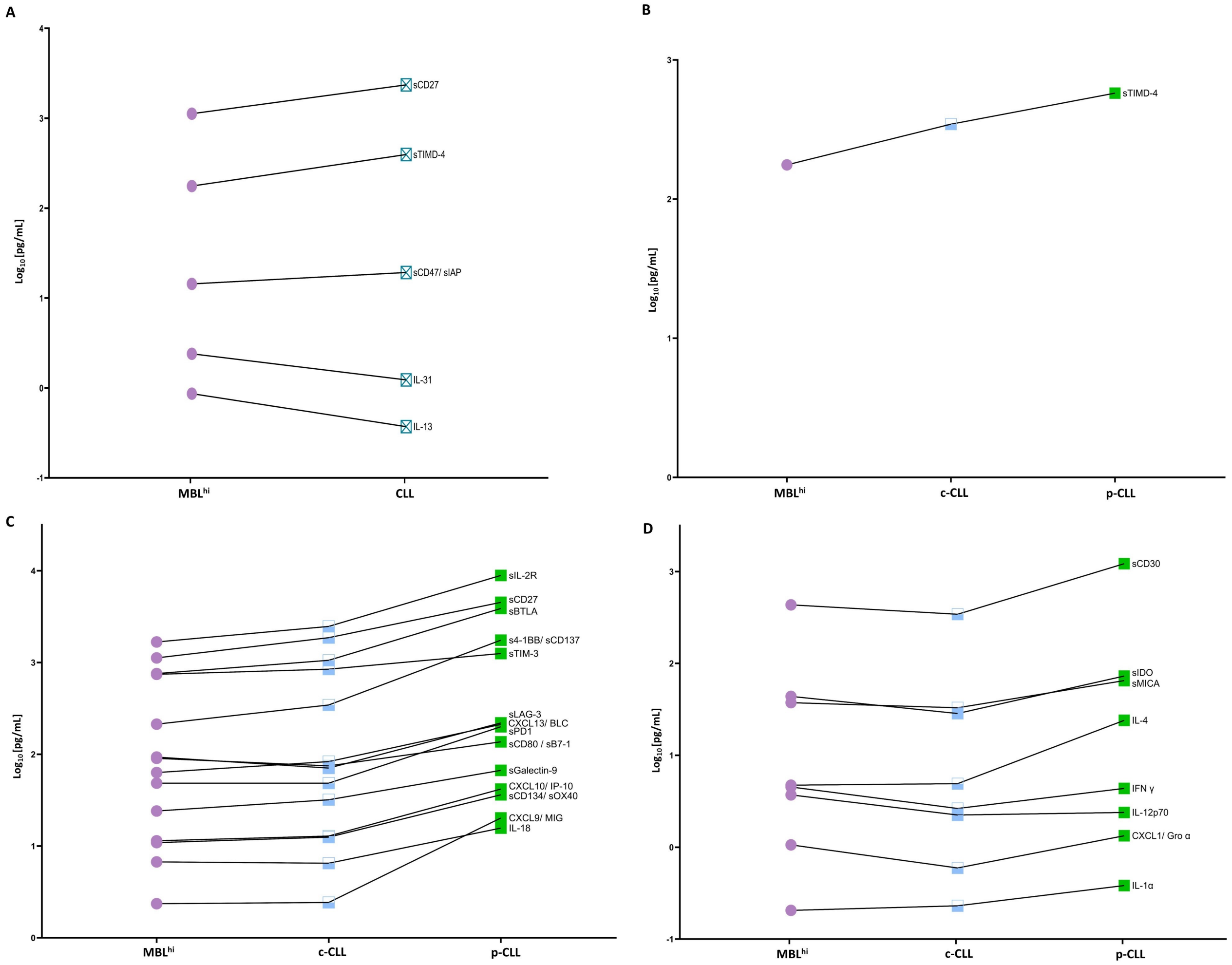

Supplementary Figure 4: Trend of significant soluble proteins after statistical analysis. Panel A.- Significant soluble proteins for comparison between monoclonal B Lymphocytosis (MBL^{hi}) and Chronic Lymphocytic Leukemia (CLL). Panel B.- Common significant soluble proteins for comparatives MBL^{hi} vs. CLL in progression (p-CLL) and MBL^{hi} vs. constant-CLL (c-CLL). Panel C.- Common significant soluble proteins for comparatives MBL^{hi} vs. p-CLL and c-CLL vs. p-CLL. Panel D.- Significant soluble proteins for comparison between c-CLL and p-CLL.

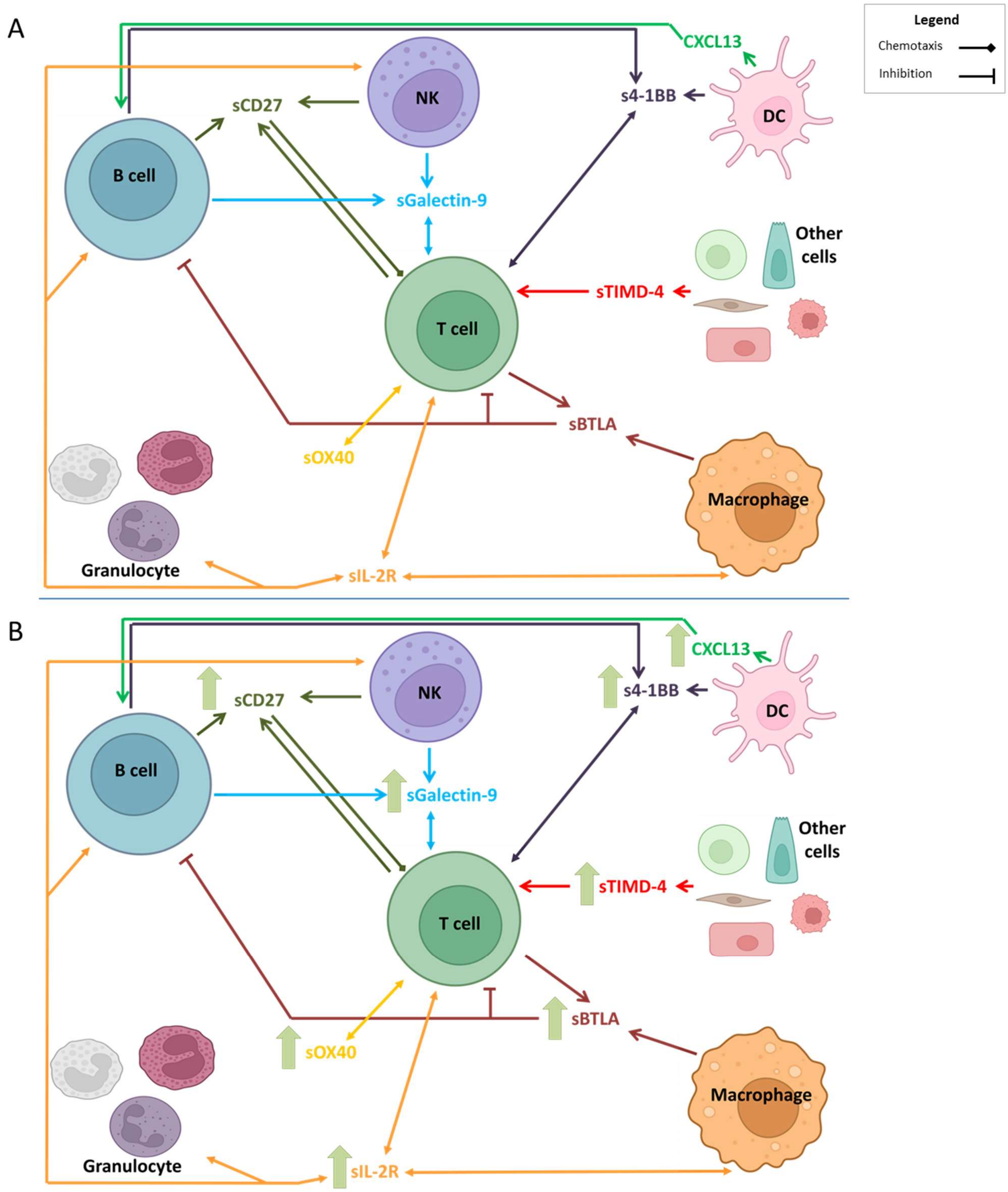

Supplementary Figure 5: TME network according to protein profiles with quantitatively increments for the diagnostic stages (monoclonal B Lymphocytosis -MBL^{hi}- and Chronic Lymphocytic Leukemia -CLL-). Panel A.- Expected model. Panel B.- Proposed model from observed datasets. (Created in Biorender.com)

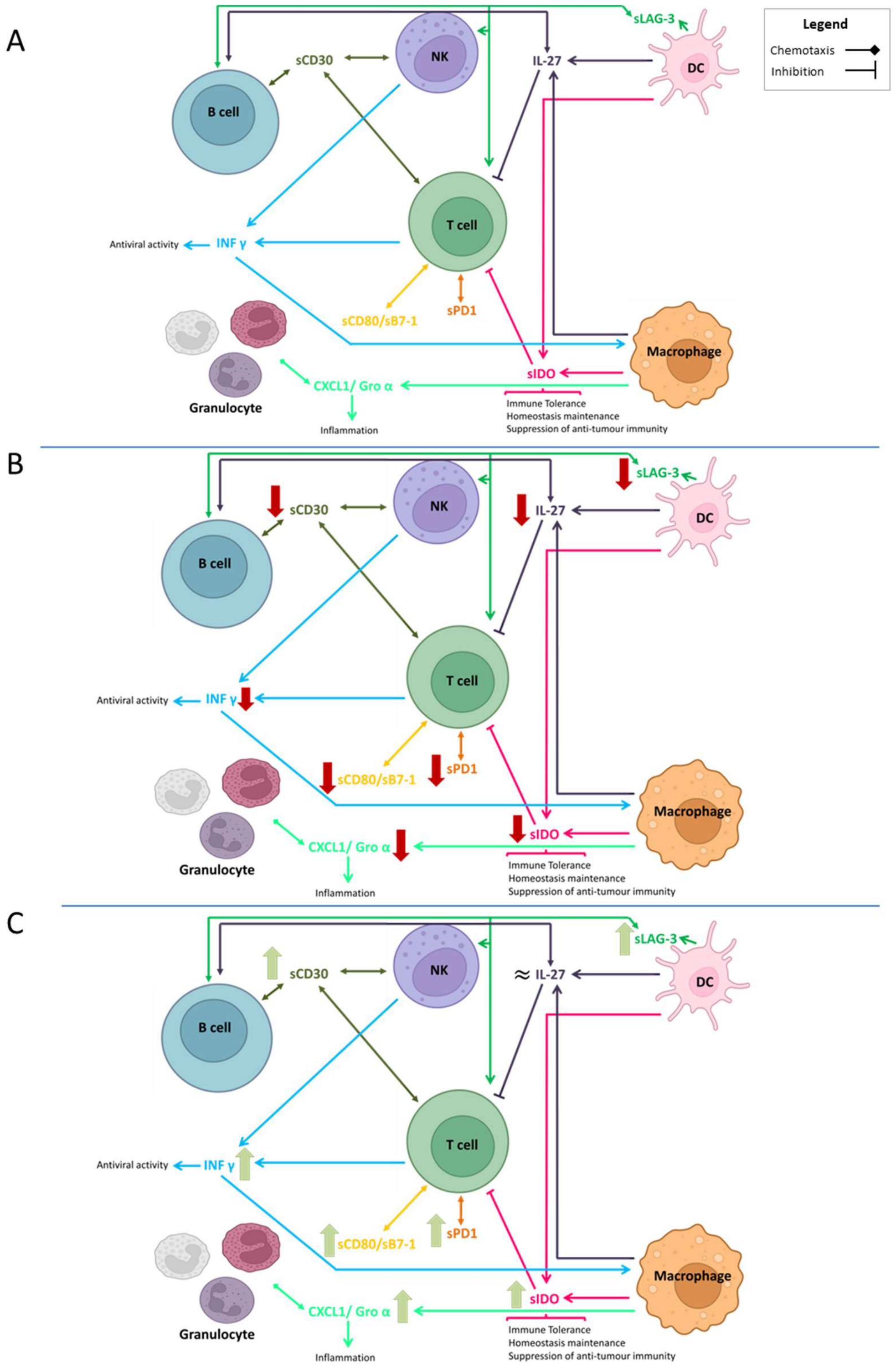

Supplementary Figure 6: TME network model proposed from protein profiles decreased according to disease evolution. Panel A.-Expected model. Panel B.-Model proposed for MBL^{hi} vs. c-CLL. Panel C.-Model proposed for c-CLL vs p-CLL. (Created in Biorender.com)

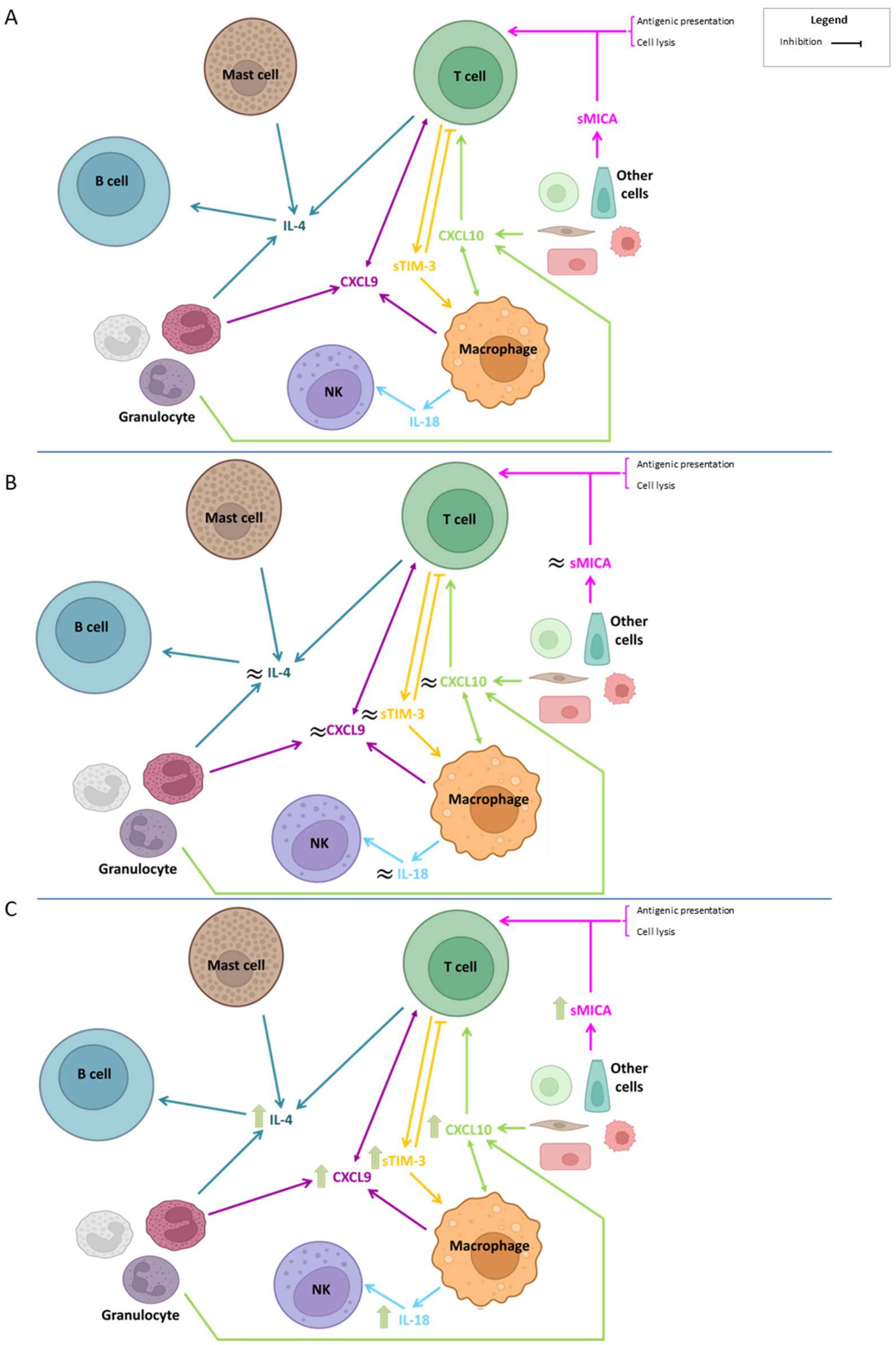

Supplementary Figure 7: Significant protein interactions with similar trend between monoclonal B Lymphocytosis (MBL^{hi}) – Stable/constant Chronic Lymphocytic Leukemia (c-CLL) and Uptrend between c-CLL -CLL in progression (p-CLL). Panel A.- Normal situation model. Panel B.- Alteration observed in the model between MBL^{hi} and c-CLL. Panel C.- Alteration observed in the model between c-CLL and p-CLL. (Created in Biorender.com)

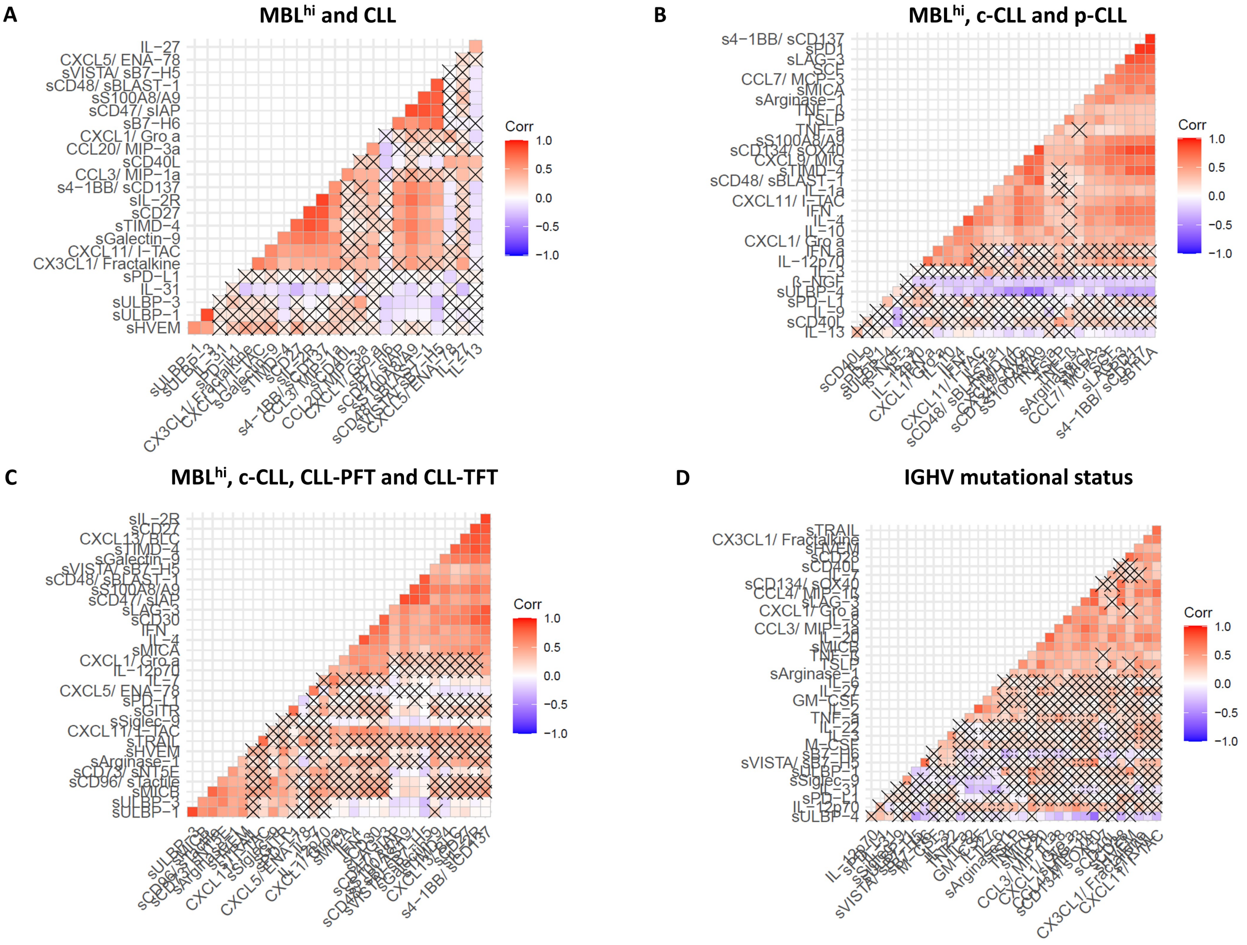

Supplementary Figure 8: Correlation matrix for each classification. Panel A.- Diagnostic group (monoclonal B Lymphocytosis -MBL^{hi} - and Chronic Lymphocytic Leukemia -CLL-). Panel B.- Diagnostic group according to progression disease (MBL^{hi}, stable/constant CLL -c-CLL- and CLL in progression -p-CLL-). Panel C.- Groups according to therapy (MBL^{hi}, c-CLL, CLL in progression previously to 1st line treatment -CLL-PFT- and CLL in progression to time from 1st line treatment –CLL-TFT-). Panel D.- Immunoglobulin Heavy-chain Variable (IGHV) mutational status. (**x meaning no correlation**)

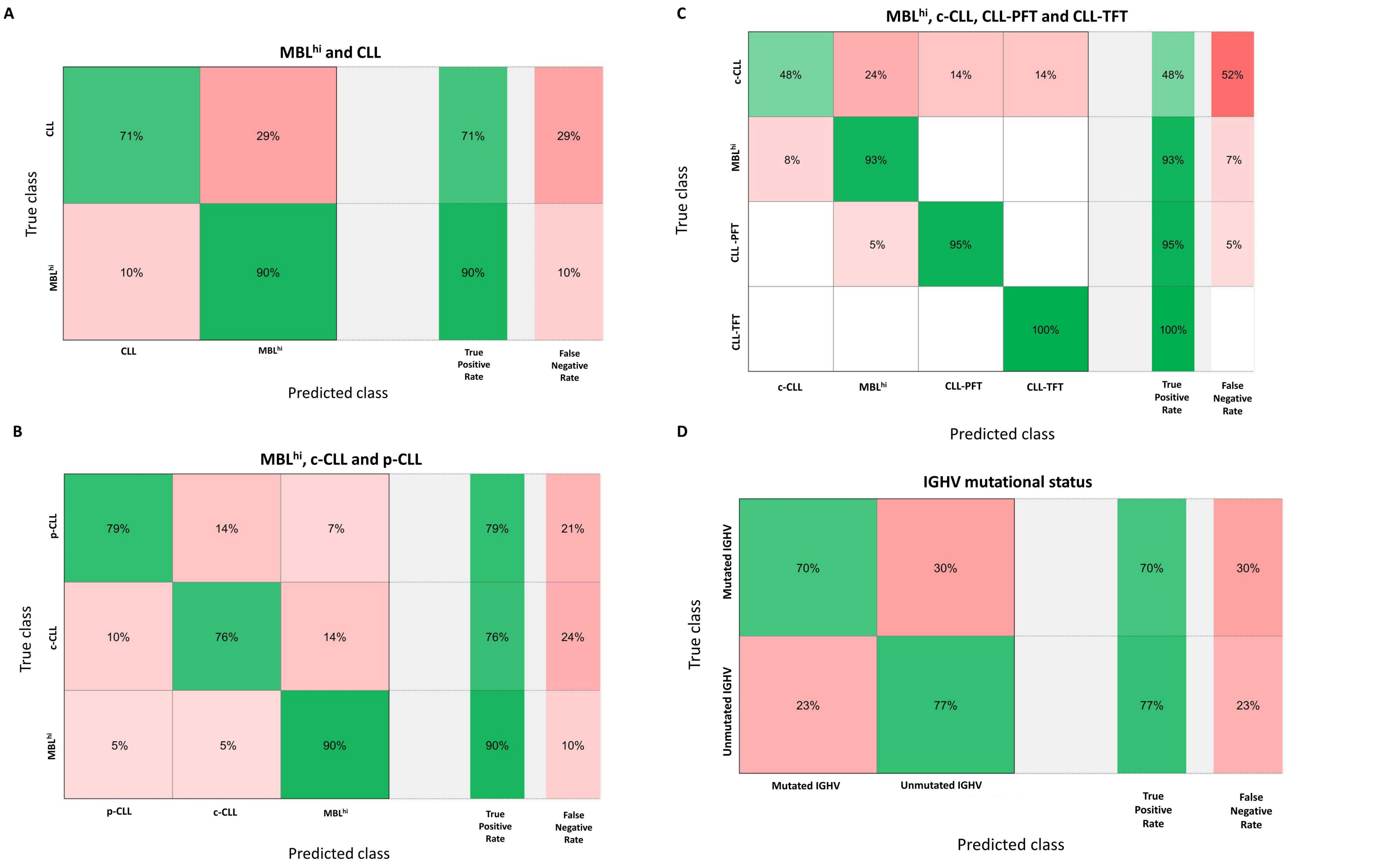

Supplementary Figure 9: Confusion matrix for decision tree of immune soluble factors. Panel A.- Results for monoclonal B Lymphocytosis (MBL^{hi}) and Chronic Lymphocytic Leukemia (CLL) decision tree. Panel B.- Results for MBL^{hi}, Stable/constant-CLL (c-CLL) and CLL in Progresion (p-CLL) decision tree. Panel C.- Results for MBL^{hi}, c-CLL, CLL in progression previously to 1st line treatment (CLL-PFT) and time from 1st line treatment (CLL-TFT). Panel D.- Results for IGHV mutational status (Mutated or Unmutated).

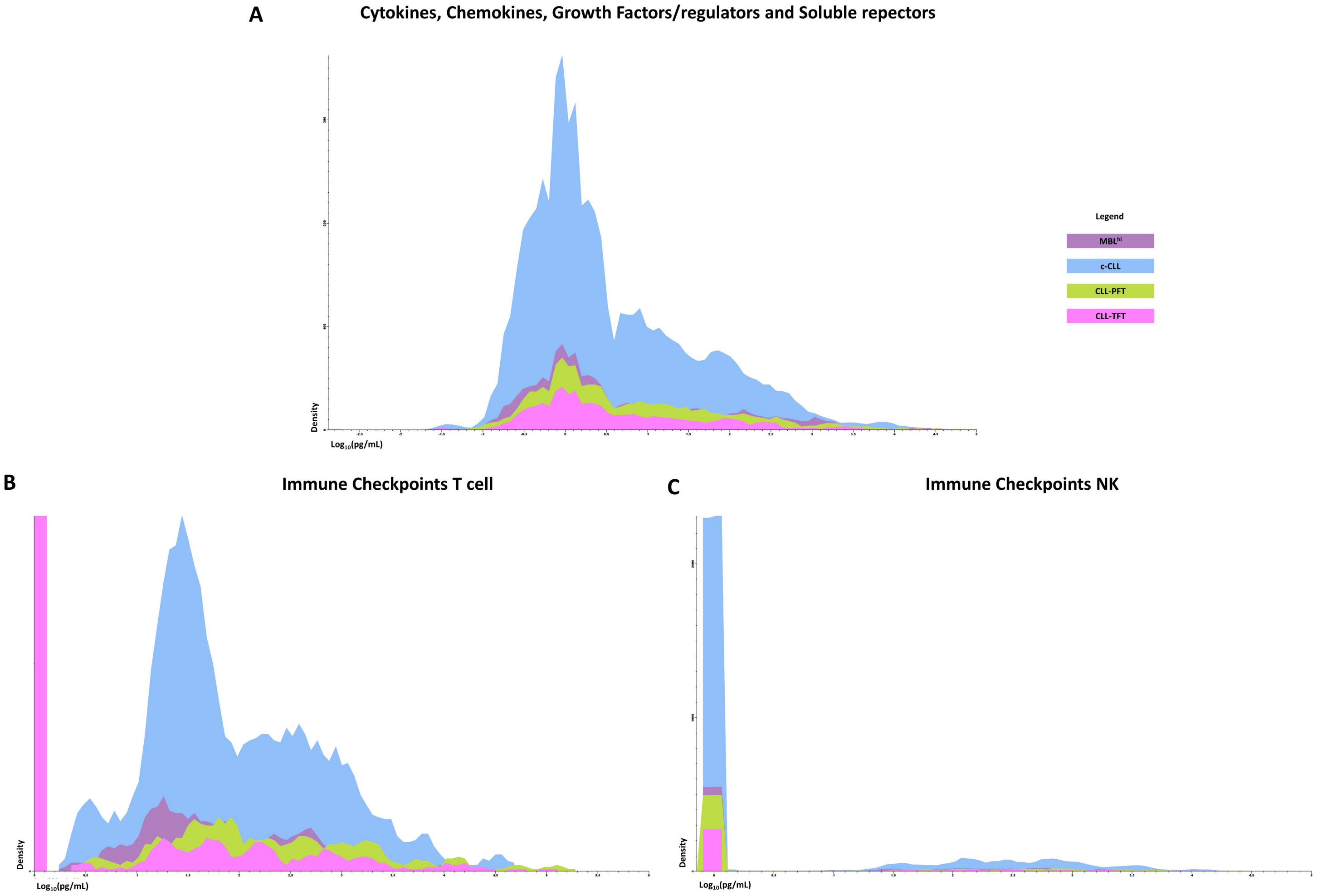

Supplementary Figure 10: Distribution data of immune soluble factors according to response to therapy (Monoclonal B Lymphocytosis –MBL^{hi}-, Stable/constant Chronic Lymphocytic Leukemia -c-CLL-, CLL in progression to previously 1st line of treatment -CLL-PFT- and CLL in progression to time from 1st line of treatment -CLL-TFT-). Panel A.- Dispersion of the average values for Cytokines, chemokines, Growth factors/regulators and soluble receptors (group 1). Panel B.- Dispersion of the average values for Immune checkpoints related with T lymphocyte (group 2). Panel C.- Dispersion of the average values for Immune checkpoints related with Natural Killer cell (group 3).

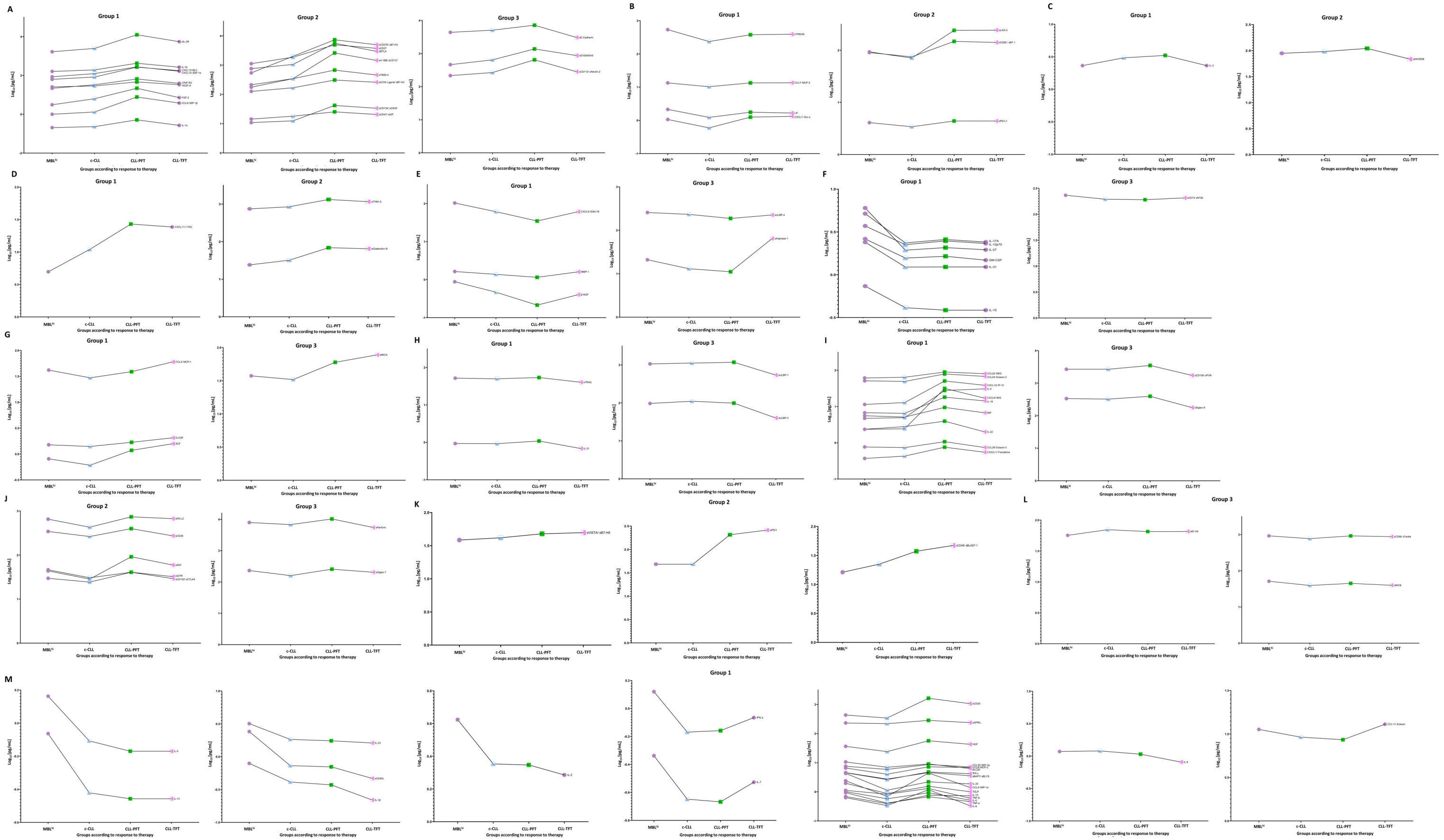

Supplementary Figure 11: Trend of immune soluble factors according to response to therapy: group 1 (cytokines, chemokines, growth factors/regulators and soluble receptors), group 2 (Immune checkpoints of T lymphocyte) and group 3 (Immune checkpoints of Natural killer cell). Panel A.- Up-Up-Down trend for three groups. Panel B.- Down-Up-Similar trend for group 1&2 of soluble proteins. Panel C.- Up-Similar-Down trend for group 1&2 of soluble proteins. Panel D.- Up-Up-Similar trend for group 1&2 of soluble proteins. Panel E.- Down-Down-Up trend for group 1&3 of soluble proteins. Panel F.- Down-Similar-Similar trend for group 1&3 of soluble proteins. Panel G.- Down-Up-Up trend for group 1&3 of soluble proteins. Panel H.- Similar-Similar-Down trend for group 1&3 of soluble proteins. Panel I.- Similar-Up-Down trend for group 1&3 of soluble proteins. Panel J.- Down-Up-Down trend for group 2&3 of soluble proteins. Panel K.- Unique trends for group 2 of soluble proteins. Panel L.- Unique trends for group 3 of soluble proteins. Panel M.- Unique trends for group 1 of soluble proteins.

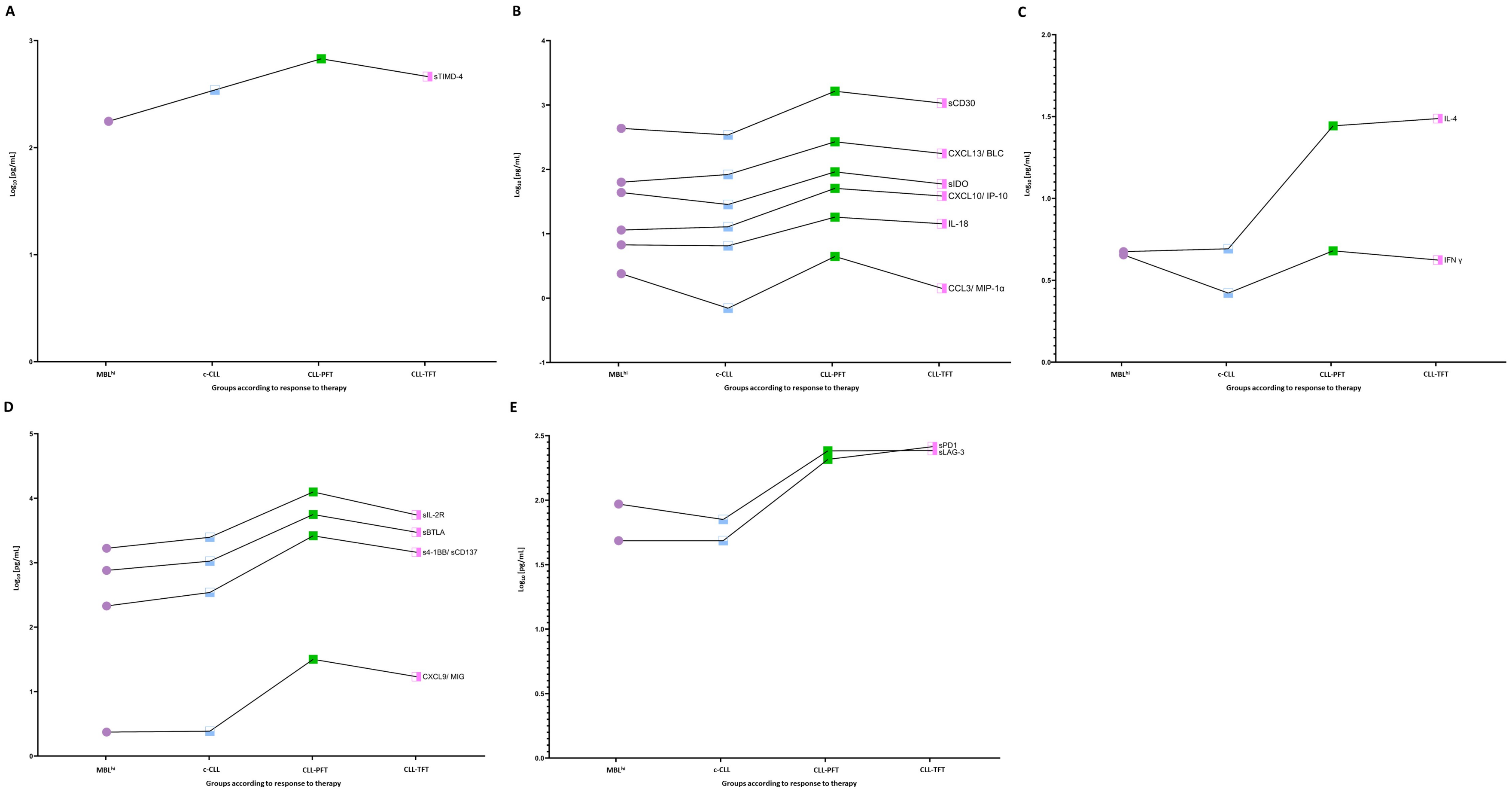

Supplementary Figure 12: Trend of significant immune soluble factors according to response to therapy after statistical analysis. Panel A.- Significant soluble proteins for comparison between Monoclonal B cell lymphocytosis (MBL^{hi}) and Chronic Lymphocytic Leukemia in progression previously to 1st line treatment (CLL-PFT). Panel B.- Significant soluble proteins for comparison between Stable/ constant-CLL (c-CLL) and CLL-PFT. Panel C.- Common significant soluble proteins for comparison c-CLL vs. CLL-PFT and c-CLL vs. CLL in progression to time from 1st line treatment (CLL-TFT). Panel D.- Common significant soluble proteins for comparison MBL^{hi} vs. CLL-PFT and c-CLL vs. CLL-PFT. Panel E.- Common significant soluble proteins for comparison MBL^{hi} vs. CLL-PFT, c-CLL vs. CLL-PFT and c-CLL vs. CLL-TFT.

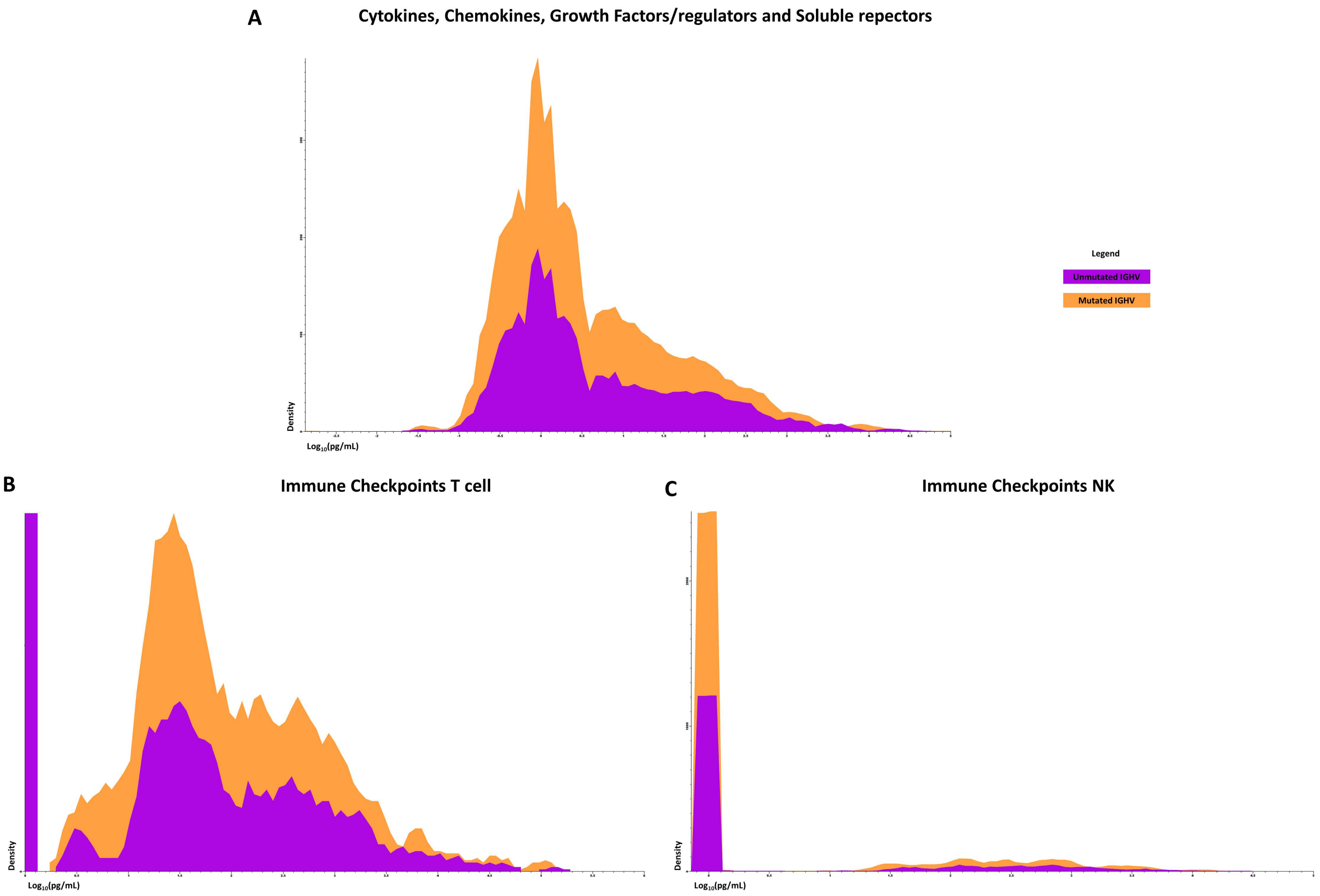

Supplementary Figure 13: Distribution data of soluble proteins for each Immunoglobulin Heavy chain Variable (IGHV) mutation status (unmutated/ mutated). Panel A.- Dispersion of the average values for Cytokines, chemokines, Growth factors/regulators and soluble receptors (group 1). Panel B.- Dispersion of the average values for Immune checkpoints related with T lymphocyte (group 2). Panel C.- Dispersion of the average values for Immune checkpoints related with Natural Killer cell (group 3).

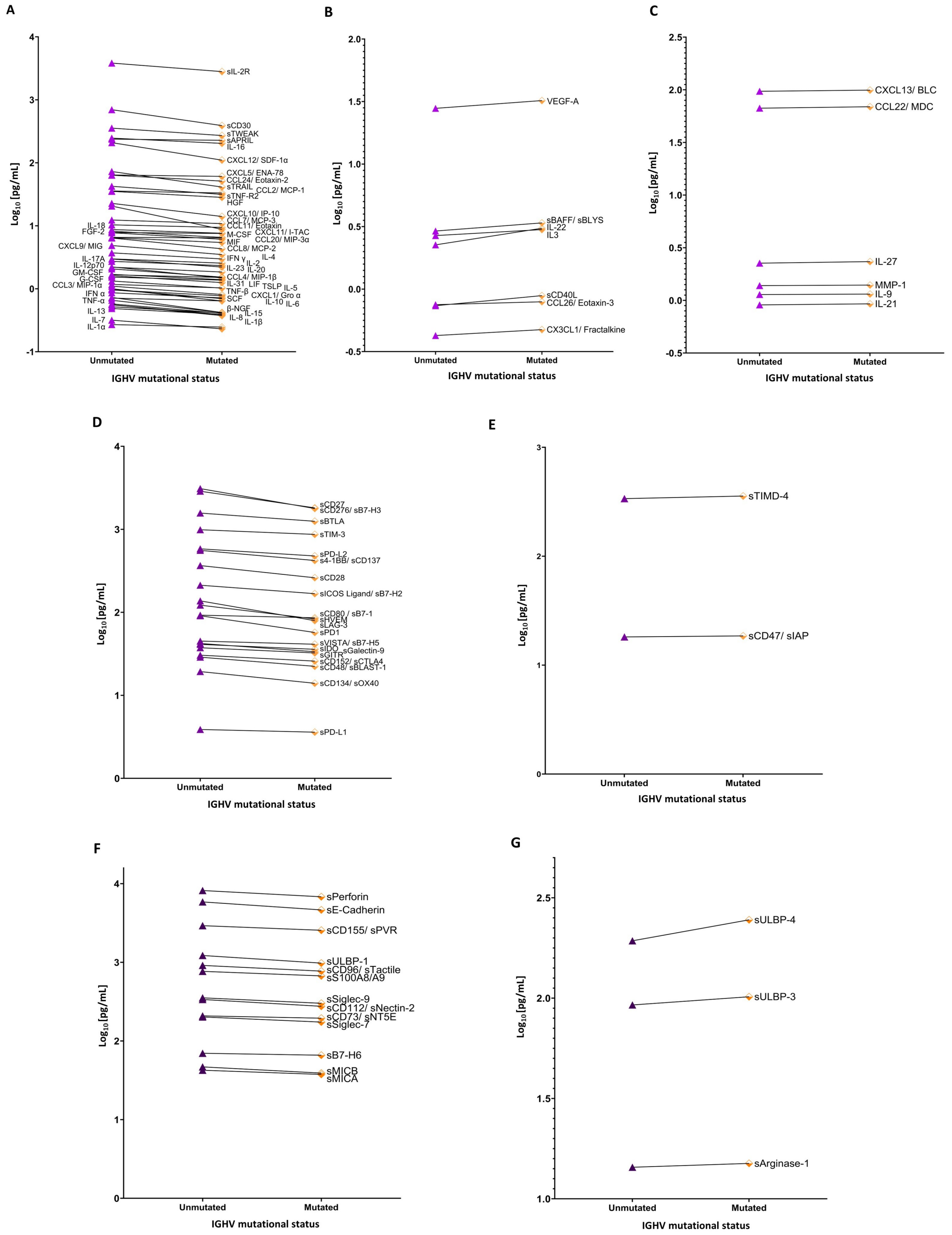

Supplementary Figure 14: Trend of soluble proteins studied for Immunoglobulin Heavy chain Variable (IGHV) mutational status. Panel A.- Decreased concentration for cytokines, chemokines, growth factors/regulators and soluble receptors. Panel B.- Increased concentration for cytokines, chemokines, growth factors/regulators and soluble receptors. Panel C.- Similar trend of concentration for cytokines, chemokines, growth factors/regulators and soluble receptors. Panel D.- Decreased concentration for Immune checkpoints of T lymphocyte. Panel E.- Similar trend of concentration for Immune checkpoints of T lymphocyte. Panel F.- Decreased concentration for Immune checkpoints of Natural killer cell. Panel G.- Increased concentration for Immune checkpoints of Natural killer cell.
